# Associations of personal care products use with reproductive outcomes of IVF/ICSI treatment

**DOI:** 10.3389/fendo.2023.1320893

**Published:** 2024-01-24

**Authors:** Qing-Chun Guo, Wen Yao, Chong Liu, Tao-Ran Deng, Juan Li, Hong-Mei Liao, Wen-Qu Tian, Yi Wang, Yao-Yao Du, Yu-Feng Li

**Affiliations:** ^1^ Department of Reproductive Medicine, Tongji Hospital, Tongji Medical College, Huazhong University of Science and Technology, Wuhan, Hubei, China; ^2^ Department of Reproductive Medicine, General Hospital of Central Theater Command, Wuhan, Hubei, China; ^3^ Department of Environmental Health, School of Public Health, Shanghai Jiao Tong University School of Medicine, Shanghai, China

**Keywords:** personal care products, cosmetics, IVF/ICSI treatment, oocyte quality, reproductive outcomes

## Abstract

**Introduction:**

Personal care products (PCPs) contain a number of endocrine-disrupting chemicals (EDCs) that could potentially affect the reproductive function in women of childbearing age. However, studies focused on the effects of PCPs use on reproductive outcomes are very limited. The current study aimed to explore the relationships between PCPs use patterns and reproductive outcomes in women undergoing *in vitro* fertilization/intracytoplasmic sperm injection (IVF/ICSI) treatment.

**Methods:**

A total of 1500 women from the Tongji Reproductive and Environmental (TREE) study between December 2018 and January 2020 were included in this study. Participants provided characteristics of PCPs use within the previous three months. Retrieved oocyte number, mature oocyte number, two distinct pronuclei (2PN) zygote number, fertilization rate, cleavage rate, blastocyst formation rate, implantation, clinical pregnancy, miscarriage, and live birth were followed up as reproductive endpoints. Generalized linear regression model was utilized to assess the associations between various categories of PCPs use and reproductive endpoints of IVF/ICSI.

**Results:**

After adjusting for relevant covariates, women who used skin care products ≥14 times per week had a reduction of 22.4% in the maturation rate (95% CI: -39.2%, -1.6%) compared to participants who did not use skin care products. After transferring fresh embryos, women who used cosmetics 1–2 times per week (adjusted OR = 2.2, 95% CI: 1.0, 4.8) or 3–7 times per week (adjusted OR = 2.5, 95% CI: 1.2, 5.2) had a higher possibility of miscarriage than those who did not use cosmetics. There was negative association between the use of gel or soap and the cleavage rate among women aged < 30 years old (*P* for interaction = 0.01). Among women with BMI ≥ 24 kg/m^2^, the use of gel or soap was negatively associated with the blastocyst formation rate (*P* for interaction = 0.04), while cosmetics use was negatively associated with the maturation rate (*P* for interaction = 0.001).

**Conclusion:**

Our findings suggest that the use of PCPs in women of reproductive age have a potential adverse impact on IVF/ICSI outcomes, particularly skin care and cosmetic products.

## 1 Introduction

Due to increasing environmental pollution and lifestyle changes associated with economic development, couples of childbearing age are facing an growing risk of infertility. Approximately 15% of couples worldwide suffer from infertility (1), which has a negative impact on their psychological and physiological health (2). As effective treatments for infertility, assisted reproductive technologies, especially *in vitro* fertilization/intracytoplasmic sperm injection (IVF/ICSI), have been chosen by many couples. According to the International Committee for Monitoring Assisted Reproductive Technologies World Report, an estimated 2.92 million assisted reproductive technology treatments were performed worldwide in 2014 (3). However, the success rate of ART has not improved. The European IVF–monitoring consortium reports that the overall clinical pregnancy rate of IVF/ICSI treatment is approximately 35% (4). Adverse environmental factors significantly impact female fertility. Therefore, it is imperative to investigate the effects of EDCs on the reproductive outcomes of IVF/ICSI.

With the improvement of sanitary and economic conditions, the use of personal care products (PCPs), including toiletries, cosmetics, and perm and hair coloring products, is gradually increasing worldwide. In 2013, the total value of China’s PCPs industry reached $2.1 billion, accounting for approximately 10% of global production (5). PCPs contain a variety of endocrine-disrupting chemicals (EDCs), including bisphenol A (BPA), phthalates (PAEs), parabens, and triclosan (TCS), which are known to affect endocrine function and impair human fertility (6). A variety of EDCs can enter the body through dermal absorption in the process of direct or indirect contact with PCPs (7). For instance, bisphenol A (BPA) and phthalates (PAEs) are commonly found in body washes and shampoos (8). They have been found to be associated with reduced semen quality, MII oocyte yield, implantation rate, and pregnancy rates (9–12). Triclosan (TCS) and triclocarban (TCC) can be used as antibacterial agent in skin care products (13, 14), and they have been reported to negatively associate with the implantation rate of IVF treatment (15, 16). Benzophenone (BPs), glycol ethers and perfluorooctanoic acid (PFOA) are frequently present in cosmetics (17–19). There’s a growing concern that these compounds might impact the onset of hormone-dependent disorders. Research has associated them with various unfavorable pregnancy outcomes, such as diminished rates of implantation, pregnancy, and live births (20, 21). p–Phenylenediamine (PPD), mainly used in hair coloring products, reduces sex hormone levels and affects oocyte quality in mice, causing abnormalities in early embryonic development (22). These studies shed light on the adverse effects of specific EDCs on reproductive health. However, the ingredients in PCPs are often a mixture of EDCs, and the real situation is more complex in daily life.

Even though the current industry regulations restrict the use of some EDCs to low levels, the possible combined effects (synergistic, inhibitory, etc.) of multiple EDCs still need to be accounted (23). However, only a limited number of studies have assessed the potential influence of PCPs use on reproductive outcomes. Therefore, we collected information on recent PCPs use among women undergoing IVF/ICSI treatment from the Tongji Reproductive and Environmental (TREE) cohort and evaluated the associations between PCPs use and eleven reproductive outcomes.

## 2 Materials and methods

### 2.1 Study participants

**Figure 1:**
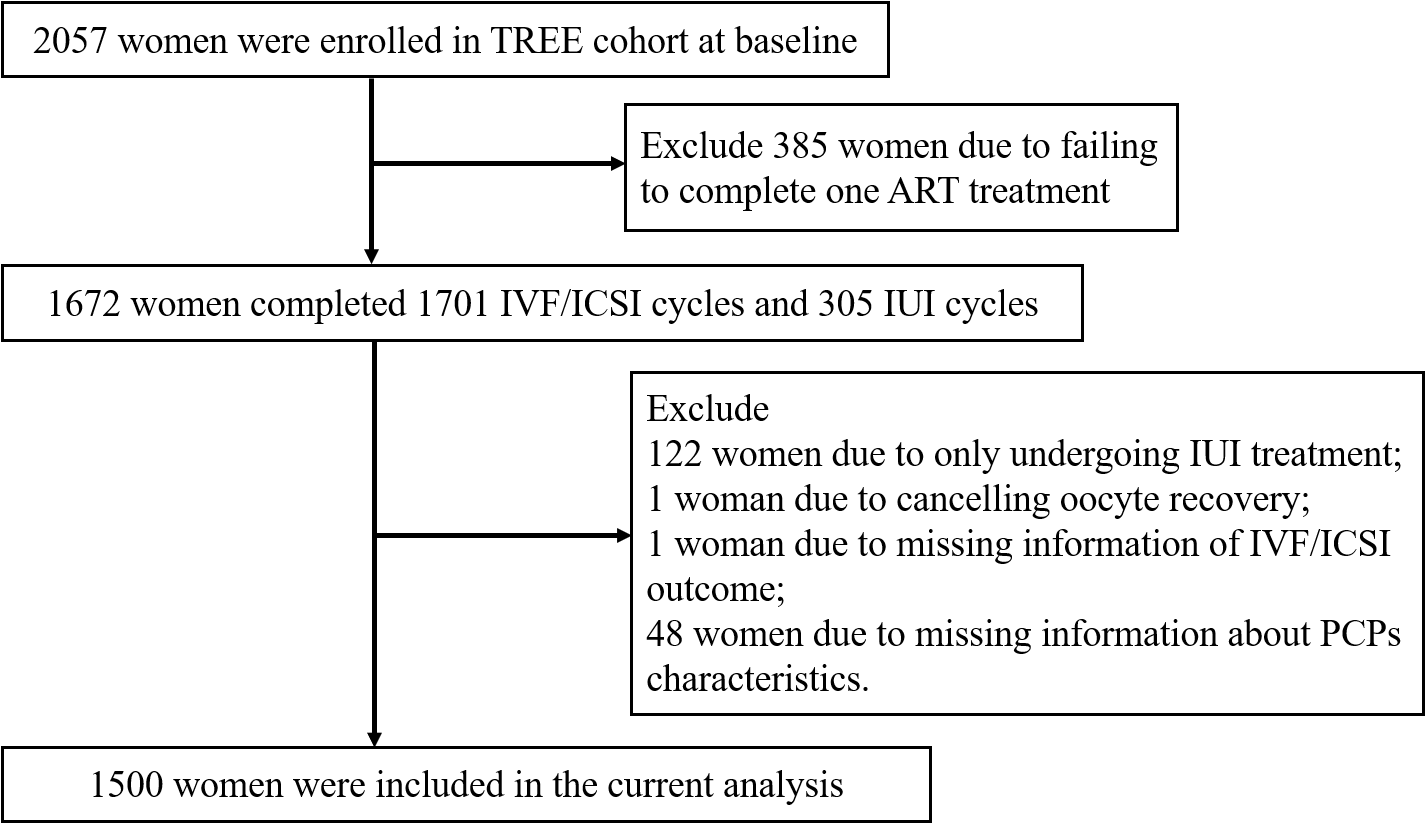
Flow chart for the study population to investigate associations between PCPs characteristics and IVF/ICSI outcomes.

All participants came from the TREE cohort. Women who were beyond 20 and seeking ART treatment at the Reproductive Medicine Center of Tongji Hospital from December 2018 to January 2020 were recruited (24). Finally, a total of 2057 couples were included in the TREE cohort. Among the participants, 1672 women underwent 1701 IVF/ICSI cycles and 305 intrauterine insemination (IUI) cycles. We further excluded 122 women due to only undergoing IUI treatment, 1 woman due to cancelling oocyte retrieval, 1 woman due to missing information about IVF/ICSI outcomes, and 48 women due to missing information about PCPs characteristics. Finally, 1500 participants were enrolled in the current analysis (**Figure 1**). Only the first oocyte aspirations were included in this study. This study was approved by the Ethics Committee of Tongji Hospital, Tongji Medical College, Huazhong University of Science and Technology. Each participant signed an informed consent form at enrollment.

### 2.2 Collection of PCPs characteristics

Upon enrollment, each participant completed a baseline questionnaire about their demographics and lifestyle. The use of PCPs was included in the questionnaire. The PCPs characteristics comprised the frequency of weekly use of gel and soap, shampoo, skin care products and cosmetics, as well as whether hair had been colored or permed in the past three months. Skin care products included toner, cream, mask, scrub, sunscreen, etc. Cosmetics included foundation, lipstick, concealer, eyebrow pencil, eye shadow, mascara, nail polish, makeup remover, etc. The frequency of gel or soap was used per week was categorized into four groups: 0, 1 to <3, 3 to <7, and ≥7 times. Shampoo use per week was categorized into four groups: ≤1, 2 to ≤3, 4 to <7 and ≥7 times. Skin care products use per week was classified into four groups: 0, 1 to <7, 7 to <14 and ≥14 times. The use of cosmetics per week was divided into four groups: 0, 1 to < 2, 3 to < 7 and ≥ 7 times.

### 2.3 Covariates

In the baseline questionnaire, sociodemographic characteristics and lifestyle characteristics were collected. Sociodemographic characteristics included ethnicity, education level, household income, usual residence, and employment status. Lifestyle characteristics included smoking, alcohol consumption, and history of use of PCPs. Smoking status was categorized into active and passive smoking. Smoking more than 100 cigarettes in a lifetime was defined as active smoking. Passive smoking exposure was defined as exposure to others’ tobacco smoke for at least 15 minutes per day on more than 1 day per week (25). Clinical characteristics were extracted from medical records, comprising age, height, weight, gravidity, parity, infertility diagnosis, controlled ovarian hyperstimulation (COH) protocols, fertilization methods, and pregnancy outcomes. Body mass index (BMI) was calculated as weight divided by the square of height. Infertility diagnoses were categorized into four groups: female factor, male factor, mixed factor, and unexplained infertility. Female factors included uterine disease, fallopian tube obstruction and ovulatory dysfunction. Male factors included testicular lesions, poor semen quality and vas deferens obstruction. The ‘mixed factors’ indicates that a couple has both male and female factors (26). Infertility without a definite diagnosis was defined as unexplained infertility. COH protocols consisted of long gonadotropin-releasing hormone (GnRH), antagonist, and other protocols (e.g., nature cycle and mild stimulation protocols).

Potential confounders were selected based on *a priori* knowledge from previous literatures. The covariates included in the final model were identified by directed acyclic graphs (DAG). In light of the notably low count of active smokers among women (n=85), passive smoking was included as a confounding factor when considering smoking status. Ultimately, all models were adjusted for the following covariates: age, BMI, educational level, household income, passive smoking and alcohol consumption (**Supplementary Figures 1, 2**). Age and BMI were treated as continuous variables. Educational level (middle school and below, high school and above), income (≤5000 yuan or >5000 yuan), passive smoking (never or ever) and alcohol consumption (never or ever) were retained as dichotomous variables.

### 2.4 IVF/ICSI procedure

IVF/ICSI procedure were conducted as previously described (27). After completion of COH protocols, once transvaginal ultrasound detected two or more follicles with a diameter of ≥ 18mm, or three or more follicles with a diameter of ≥ 17mm, HCG is administered to induce ovulation. Then oocytes and embryos were cultured in sequential embryo culture media. The number of two distinct pronuclei (2PN) zygotes, number of cleavage-stage embryo, and rates of fragmentation were recorded. High-quality embryos were selected to be transferred or cryopreserved, while remaining embryos were cultured to the blastocyst stage before cryopreservation.

### 2.5 Reproductive outcomes

The following parameters served as intermediate endpoints for IVF/ICSI procedures: the number of retrieved oocytes, number of mature oocytes, maturation rate, number of normal fertilized oocytes, fertilization rate, cleavage rate and blastocyst formation rate. Mature oocytes were defined as those that had expelled the first polar body and reached the MII stage. The maturation rate was determined by the ratio of mature oocytes to the total number of oocytes retrieved. After fertilization, zygotes with two distinct pronuclei were referred as 2PN zygotes. The fertilization rate was calculated by dividing the number of 2PN zygotes by the number of mature oocytes (28). The cleavage rate is the ratio of the number of cleavage-stage embryos divided by the number of 2PN zygotes (29). The blastocyst formation rate was defined as the number of blastocysts on day five divided by the number of embryos in culture (30).

Pregnancy outcomes included implantation, clinical pregnancy, miscarriage, and live birth. Implantation was defined as a serum human chorionic gonadotropin (HCG) level exceeding 10 mIU/mL approximately 14 days after embryo transfer. Clinical pregnancy was identified when the fetal heartbeat and the fetal sac were observed by ultrasound 28 days after embryo transfer. Loss of pregnancy at less than 28 weeks of gestation was termed miscarriage. A live birth was defined as the delivery of a newborn after 28 weeks of gestation (31).

### 2.6 Statistical analysis

The mean ± standard deviation (SD) or n (%) was used to describe the demographic, clinical, and PCPs characteristics of all participants. Generalized linear models were used to assess the associations between PCPs characteristics and IVF/ICSI outcomes, using PCPs characteristics as categorical variables. The counts of retrieved oocytes, mature oocytes, and 2PN zygotes were analyzed using the quasi-Poisson distribution and the log-link function. Maturation and fertilization rates were assessed using the quasi-binomial distribution and logit function. After obtaining the regression coefficient, the percentage change was calculated using the following formula: [exp () - 1] × 100%. For implantation, clinical pregnancy, miscarriage, and live birth, binomial distribution and logit function were used. Odds ratios (OR) and 95% CI are used to describe the results. Data with missing values were deleted (all < 5%) (32).

Stratified analyses were performed with the PCPs characteristics as continuous variables. In this study, stratified analyses were specifically conducted for age and BMI. Interaction terms involving the use of different PCPs with age and BMI were included in the GLM described above to validate any interaction effects. In addition, sensitivity analyses were performed to test the robustness of the results. First, crude associations between PCPs characteristics and IVF/ICSI endpoints were analyzed. Second, we excluded women with a diagnosis of PCOS or endometriosis and reanalyzed the data. Finally, association analyses were performed for participants without male-factor infertility. R software (version 4.2.1: The R Project for Statistical Computing, Austria) was used to analyze the data. A *P*-value of less than 0.05 was regarded as statistically significant.

## 3 Results

### 3.1 Population characteristics

A total of 1500 women were included in this study. **Table 1** lists the demographic, clinical, and PCPs characteristics of all participants. The average age of the participants was 30.9 years, and the mean BMI was 22.1 kg/m^2^. The majority of participants were of Han ethnicity (96.1%). Most women came from rural areas (64.4%) and were unemployed (51.5%). A total of 94.3% of the participants had never smoked, 76.9% had never consumed alcohol, and 55.3% had never been pregnant. A total of 852 participants (56.8%) were diagnosed with infertility due to female factors. Regarding the use of PCPs, more than half of the women used gel or soap >7 times per week (50.3%). A total of 54.7% of participants used shampoo 4 to <7 times a week. Most participants used skin care products 7 to <14 times per week (45.2%) and did not use make-up products (43.2%). In the past three months, 83.7% of the women had not permed or dyed their hair.

**Table 1:** Demographic, clinical and PCPs characteristics of women included in this study (n=1500).

Characteristics	Mean ± SD or n (%)
Demographic characteristics	
Age (years)	30.9 ± 4.8
BMI (kg/m^2^)	22.1 ± 3.1
Ethnicity	
Han	1441 (96.1%)
Others	59 (3.9%)
Education level	
Middle school and below	603 (40.2%)
High school and above	897 (59.8%)
Income (yuan)	
≤5000	763 (50.9%)
>5000	737 (49.1%)
Permanent residence	
Urban area	534 (35.6%)
Rural area	966 (64.4%)
Working status	
Unemployed	773 (51.5%)
Employed	692 (46.1%)
Smoking status	
Never	1415 (94.3%)
Ever	85 (5.7%)
Passive smoking	
Never	775 (51.7%)
Ever	725 (48.3%)
Alcohol consumption	
Never	1154 (76.9%)
Ever	346 (23.1%)
Clinical characteristics	
Number of retrieved oocytes	12.4 ± 7.4
Number of mature oocytes	10.3 ± 6.5
Maturation rate (%)	84.9 ± 16.8
Normal fertilized oocytes (2PN)	7.4 ± 5.1
Fertilization rate (%)	70.0 ± 22.6
Cleavage rate (%)	97.0 ± 10.8
Good–quality embryos on day 3	3.9 ± 3.4
Blastocyst formation rate	37.3 ± 29.3
Gravidity	
0	829 (55.3%)
≥1	671 (44.7%)
Parity	
0	1241 (82.7%)
≥1	259 (17.3%)
Infertility diagnosis	
Female factors	852 (56.8%)
Male factors	167 (11.1%)
Mix factors	356 (23.7%)
Unexplained	125 (8.3%)
COH protocols	
Long GnRH	917 (61.1%)
Antagonist	442 (29.5%)
Others	141 (9.4%)
Fertilization methods	
IVF	1016 (67.7%)
ICSI	467 (31.1%)
Implantation	
No	355 (26.0%)
Yes	1009 (74.0%)
Clinical pregnancy	
No	547 (36.5%)
Yes	953 (63.5%)
Live birth	
No	630 (42.0%)
Yes	870 (58.0%)
PCPs characteristics	
Use of gel or soap (times per week)	
0	96 (6.4%)
	200 (13.3%)
3~<7	429 (28.6%)
≥7	755 (50.3%)
Use of shampoo (times per week)	
≤1	75 (5.0%)
2~≤3	495 (33.0%)
4~<7	820 (54.7%)
≥7	107 (7.1%)
Use of skin care products (times per week)	
0	170 (11.3%)
1~<7	266 (17.7%)
7~<14	678 (45.2%)
≥14	363 (24.2%)
Use of cosmetics (times per week)	
0	648 (43.2%)
1~≤2	210 (14.0%)
3~<7	252 (16.8%)
≥7	366 (24.4%)
Dyeing or perming hair in past 3 months	
No	1256 (83.7%)
Yes	244 (16.3%)

A total of 35 women were missing for working status, 20 for use of gel or soap, 3 for use of shampoo, 23 for use of skin care products, 24 for use of cosmetics.

BMI, body mass index; SD, standard deviation; 2PN, two distinct pronuclei; COH, controlled ovarian hyperstimulation; GnRH, gonadotrophin–releasing hormone; IVF, in vitro fertilization; ICSI, intracytoplasmic sperm injection; PCPs, personal care products.

### 3.2 Clinical characteristic

**Table 1** provides the cycle-specific characteristics and clinical outcomes. The long GnRH protocol was used in 917 (61.1%) of the 1500 IVF/ICSI cycles, and the antagonist protocol was used in 442 cycles (29.5%). For the insemination method, IVF was applied in 1016 cycles (67.7%), and ICSI was applied in 467 cycles (31.1%). In 1470 cycles, embryo transfer was carried out, of which the percentages of implantation, clinical pregnancy and live birth were 74.0%, 63.5% and 58.0%, respectively.

### 3.3 Associations between PCPs characteristics and IVF/ICSI outcomes

**Table 2** illustrates the relationships between PCPs characteristics and IVF/ICSI intermediate endpoints in the first oocyte aspirations. Compared to participants who did not use skin care products, women who used skin care products ≥14 times per week had a 22.4% (95% CI: –39.2%, –1.6%) decrease in maturation rate. In addition, the participants who used gel or soap ≥7 times per week had 3.2% (95% CI: –14.1%, 9.4%), 6.2% (95% CI: –17.1%, 6.5%), 12.3% (95% CI: –34.1%, 15.1%), and 4.7% (95% CI: –17.0%, 10.0%) decreases in the number of retrieved oocytes, MII oocytes, maturation rate, and 2PN zygotes, respectively. However, these associations were not statistically significant.

**Table 2:** Associations between PCPs characteristics and IVF/ICSI parameters in the TREE study (n=1500).

Characteristics (times per week)	Number of retrieved oocytes	Number of mature oocytes	Maturation rate	Number of 2PN zygotes	Fertilization rate	Cleavage rate	Blastocyst formation rate
	% Change (95% CI)	% Change (95% CI)	% Change (95% CI)	% Change (95% CI)	% Change (95% CI)	% Change (95% CI)	% Change (95% CI)
Use of gel or soap							
0	Ref	Ref	Ref	Ref	Ref	Ref	Ref
1~<3	–1.7 (–14.4, 13.2)	–5.2 (–17.9, 9.8)	–12.3 (–36.4, 19.9)	–5.0 (–19.2, 12.0)	1.1 (–22.0, 30.6)	20.5 (–54.3, 195.0)	18.1 (–17.7, 70.4)
3~<7	–0.39 (–12.0, 13.1)	–4.4 (–15.9, 9.1)	–17.8 (–38.8, 8.9)	–2.7 (–15.8, 12.9)	9.6 (–13.6, 38.4)	47.6 (–40.8, 227.7)	25.7 (–9.3, 75.5)
≥7	–3.2 (–14.1, 9.4)	–6.2 (–17.1, 6.5)	–12.3 (–34.1, 15.1)	–4.7 (–17.0, 10.0)	12.1 (–10.8, 40.1)	–5.3 (–60.0, 93.4)	–1.7 (–28.3, 35.9)
Use of shampoo							
≤1	Ref	Ref	Ref	Ref	Ref	Ref	Ref
2~≤3	6.9 (–7.2, 23.7)	2.6 (–11.3, 19.5)	–10.5 (–35.4, 21.9)	1.7 (–13.6, 20.6)	–1.0 (–24.4, 28.6)	60.6 (–31.8, 236.2)	25.8 (–13.5, 85.1)
4~<7	2.4 (–10.8, 18.2)	–1.0 (–14.2, 14.9)	–8.2 (–33.4, 24.1)	–2.3 (–16.7, 15.4)	4.8 (–19.5, 35.4)	80.0 (–22.3, 266.8)	23.3 (–14.5, 80.3)
≥7	3.0 (–13.3, 22.7)	–0.94 (–17.3, 18.8)	–8.6 (–38.0, 33.6)	–4.1 (–21.6, 17.6)	–0.59 (–28.0, 36.9)	11.3 (–57.9, 182.3)	35.1 (–13.7, 113.2)
Use of skin care products							
0	Ref	Ref	Ref	Ref	Ref	Ref	Ref
1~<7	6.6 (–4.8, 19.6)	4.1 (–7.5, 17.5)	–21.4 (–38.9, 0.77)	8.8 (–4.8, 24.6)	9.0 (–11.6, 34.1)	–18.4 (–65.6, 81.3)	7.0 (–20.6, 44.5)
7~<14	6.6 (–3.7, 18.2)	5.7 (–4.9, 17.9)	–8.9 (–27.7, 14.0)	8.6 (–3.7, 22.7)	10.0 (–8.6, 32.1)	–36.0 (–70.9, 26.1)	2.1 (–21.8, 33.8)
≥14	9.3 (–2.0, 22.0)	7.7 (–3.9, 20.8)	**–22.4 (–39.2, –1.6)** *	8.9 (–4.2, 24.1)	7.2 (–12.2, 30.6)	–23.1 (–66.6, 62.6)	13.0 (–14.9, 50.6)
Use of cosmetics							
0	Ref	Ref	Ref	Ref	Ref	Ref	Ref
1~≤2	4.6 (–4.1, 14.0)	4.6 (–4.6, 14.5)	4.9 (–13.8, 28.2)	1.2 (–8.8, 12.1)	–9.7 (–23.6, 6.8)	–6.0 (–46.5, 73.1)	14.3 (–9.2, 43.6)
3~<7	–1.5 (–9.5, 7.0)	–0.52 (–8.9, 8.6)	10.0 (–8.7, 32.9)	0.62 (–8.8, 10.8)	9.6 (–6.6, 28.8)	62.1 (–13.6, 230.1)	–1.6 (–21.1, 22.5)
≥7	3.4 (–4.0, 11.3)	4.0 (–3.8, 12.4)	3.9 (–11.8, 22.7)	4.3 (–4.4, 13.7)	3.3 (–10.3, 19.0)	–21.6 (–50.6, 25.9)	12.6 (–7.4, 36.8)
Dyeing or perming hair in past 3 months							
No	Ref	Ref	Ref	Ref	Ref	Ref	Ref
Yes	1.1 (–6.6, 9.3)	–0.91 (–8.9, 7.6)	–6.3 (–20.9, 11.4)	0.60 (–8.4, 10.2)	–2.1 (–15.5, 13.7)	–17.9 (–49.4, 39.7)	1.0 (–18.1, 24.1)

Generalized linear regression models were adjusted for age, BMI, passive smoking, alcohol consumption, education level and income. Bold indicates that the item has statistical significance. *P < 0.05.

PCPs, personal care products; IVF, in vitro fertilization; ICSI, intracytoplasmic sperm injection; 2PN, two distinct pronuclei; BMI, body mass index; Ref, reference.

Embryo transfer was performed in 1,364 cycles. **Table 3** illustrates the associations between PCPs characteristics and pregnancy outcomes. A null association was found between PCPs use and implantation, clinical pregnancy, miscarriage, and live birth.

**Table 3:** Associations between PCPs characteristics and IVF/ICSI pregnancy outcomes among 1364 cycles with embryos transferred.

Characteristics (times per week)	Implantation	Clinical pregnancy	Miscarriage	Live birth
	OR (95% CI)	OR (95% CI)	OR (95% CI)	OR (95% CI)
Use of gel or soap				
0	Ref	Ref	Ref	Ref
1~<3	1.3 (0.76, 2.2)	1.4 (0.81, 2.3)	1.2 (0.46, 3.6)	1.3 (0.75, 2.1)
3~<7	1.3 (0.79, 2.1)	1.5 (0.92, 2.4)	0.91 (0.38, 2.6)	1.4 (0.90, 2.3)
≥7	1.1 (0.67, 1.7)	1.1 (0.70, 1.8)	0.64 (0.27, 1.8)	1.2 (0.77, 1.9)
Use of shampoo				
≤1	Ref	Ref	Ref	Ref
2~≤3	1.2 (0.71, 2.1)	1.1 (0.66, 1.9)	0.47 (0.20, 1.3)	1.4 (0.82, 2.4)
4~<7	1.2 (0.70, 2.0)	1.1 (0.67, 1.9)	0.48 (0.20, 1.3)	1.4 (0.83, 2.4)
≥7	1.3 (0.67, 2.5)	1.1 (0.55, 2.0)	0.53 (0.15, 1.8)	1.3 (0.67, 2.4)
Use of skin care products				
0	Ref	Ref	Ref	Ref
1~<7	0.86 (0.55, 1.3)	0.96 (0.63, 1.5)	0.63 (0.27, 1.5)	1.1 (0.73, 1.7)
7~<14	1.2 (0.82, 1.8)	1.2 (0.80, 1.7)	0.60 (0.30, 1.3)	1.3 (0.91, 1.9)
≥14	1.1 (0.74, 1.7)	1.2 (0.77, 1.8)	0.71 (0.33, 1.6)	1.3 (0.85, 1.9)
Use of cosmetics				
0	Ref	Ref	Ref	Ref
1~≤2	0.93 (0.65, 1.3)	0.92 (0.66, 1.3)	0.78 (0.34, 1.6)	0.99 (0.71, 1.4)
3~<7	0.88 (0.64, 1.2)	0.98 (0.71, 1.4)	1.5 (0.79, 2.7)	0.89 (0.65, 1.2)
≥7	1.02 (0.76, 1.4)	1.1 (0.82, 1.5)	0.83 (0.44, 1.5)	1.1 (0.85, 1.5)
Dyeing or perming hair in past 3 months				
No	Ref	Ref	Ref	Ref
Yes	1.1 (0.79, 1.5)	1.1 (0.86, 1.6)	1.1 (0.64, 2.1)	1.1 (0.82, 1.5)

Generalized linear regression models were adjusted for age, BMI, passive smoking, alcohol consumption, education level and income.

PCPs, personal care products; IVF, in vitro fertilization; ICSI, intracytoplasmic sperm injection; OR, odd ratio; BMI, body mass index; Ref, reference.

### 3.4 Stratified analyses

**Figure 2:**
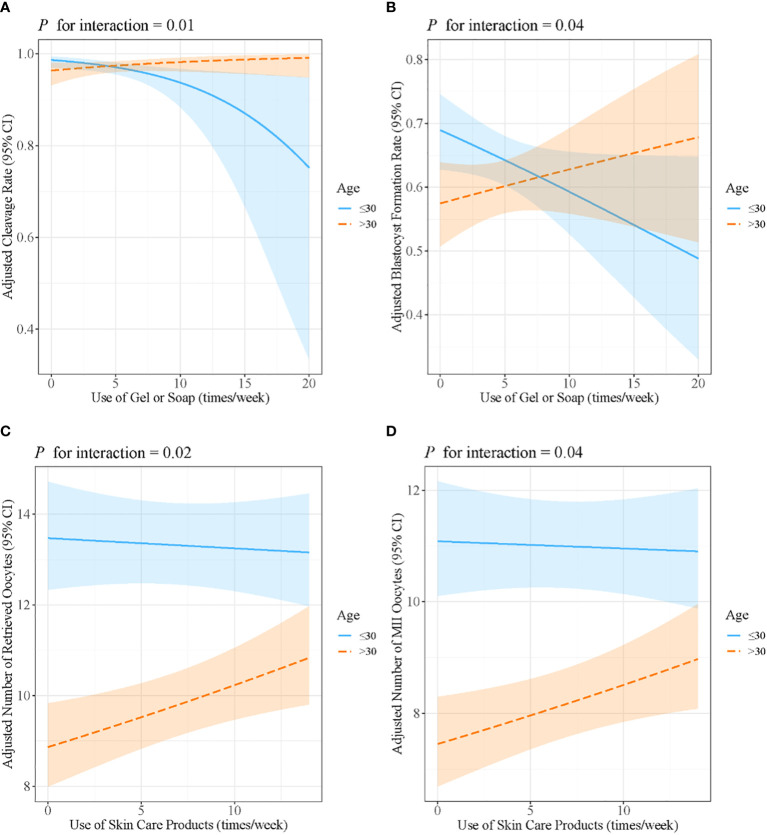
Associations between the use of gel or soap and **(A)** cleavage rate and **(B)** blastocyst formation rate, stratified by maternal age. Associations between the use of skin care products and **(C)** the number of retrieved oocytes and **(D)** the number of MII oocytes, stratified by maternal age. Models were adjusted for age, BMI, passive smoking, alcohol consumption, education level and income. MII, metaphase II; BMI, body mass index.

Age and BMI were stratified to further analyze the interaction effects. The results are presented in **Supplementary Tables 1, 2**. Age was found to modify the associations of the use of gel or soap with the cleavage rate (*β*: −0.16, 95% CI: −0.27, −0.06, *P* = 0.004 for women younger than 30 years; *β*: 0.07, 95% CI: −0.05, 0.20, *P* = 0.23 for women aged over 30 years; *P* for interaction = 0.01) and the blastocyst formation rate (*β*: −0.04, 95% CI: −0.08, −0.002, *P* = 0.07 for women younger than 30 years; *β*: 0.02, 95% CI: −0.03, 0.07, *P* = 0.39 for women aged over 30 years; *P* for interaction = 0.04). Age also modified the association of the use of skin care products with the retrieved oocyte number (*β*: −0.002, 95% CI: −0.01, 0.01, *P* = 0.70 for women younger than 30 years; *β*: 0.01, 95% CI: 0.003, 0.02, *P* = 0.01 for women aged over 30 years; *P* for interaction = 0.02) and the number of MII oocytes (*β*: −0.001, 95% CI: −0.01, −0.01, *P* = 0.78 for women younger than 30 years; *β*: 0.01, 95% CI: 0.002, 0.02, *P* = 0.02 for women aged over 30 years; *P* for interaction = 0.04) (**Figure 2**).

**Figure 3:**
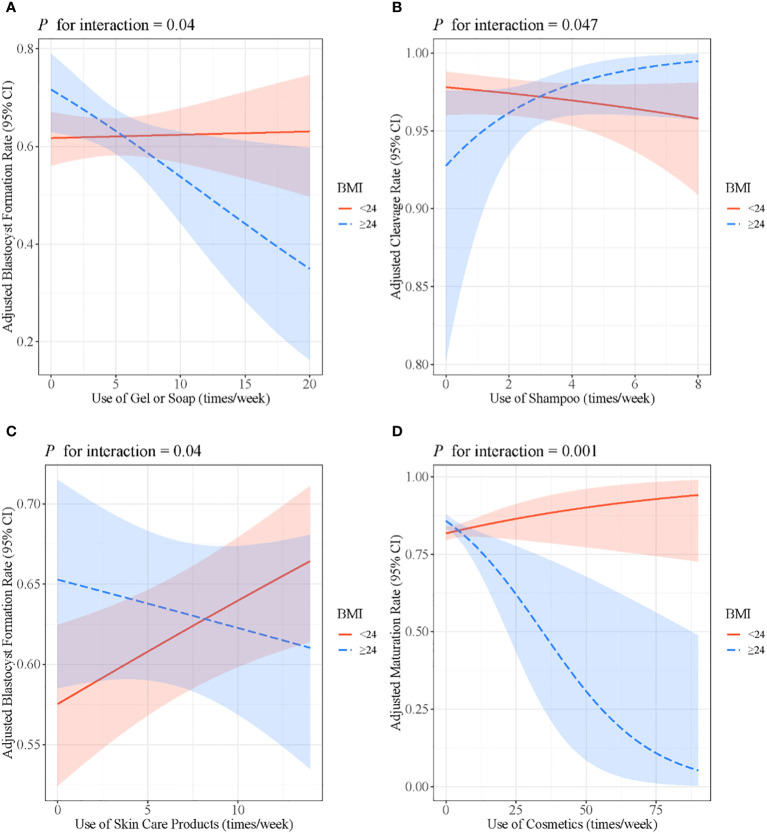
**(A)** Associations between the use of gel or soap and blastocyst formation rate stratified by BMI. **(B)** Associations between the use of shampoo and cleavage rate stratified by BMI. **(C)** Associations between the use of skin care products and blastocyst formation rate stratified by BMI. **(D)** Associations between the use of cosmetics and maturation rate stratified by BMI. Models were adjusted for age, BMI, passive smoking, alcohol consumption, education level and income. BMI, body mass index.

The association between the use of gel or soap and the blastocyst formation rate was only significant among women with BMI ≥24 kg/m^2^ (*β*: 0.002, 95% CI: −0.03, 0.04, *P* = 0.92 for women <24 kg/m^2^; *β*: −0.08, 95% CI: −0.14, −0.01, *P* = 0.02 for women ≥24 kg/m^2^; *P* for interaction = 0.04). In addition, BMI modified the association between the use of shampoo and cleavage rate (*β*: −0.08, 95% CI: −0.21, 0.07, *P* = 0.27 for women <24 kg/m^2^; *β*: 0.36, 95% CI: 0.03, 0.75, *P* = 0.05 for women ≥24 kg/m^2^; *P* for interaction = 0.047), as well as the association between the use of skin care products and the blastocyst formation rate (*β*: 0.03, 95% CI: 0.01, 0.05, *P* = 0.01 for women <24 kg/m^2^; *β*: −0.02, 95% CI: −0.05, 0.04, *P* = 0.28 for women ≥24 kg/m^2^; *P* for interaction = 0.04). The positive association of the use of cosmetics with the maturation rate only existed in women with BMI ≥24 kg/m^2^ (*β*: 0.01, 95% CI: −0.004, 0.04, *P* = 0.16 for women <24 kg/m^2^; *β*: −0.05, 95% CI: −0.09, 0.02, *P* = 0.001 for women ≥24 kg/m^2^; *P* for interaction = 0.001) (**Figure 3**).

### 3.5 Sensitivity analysis

The results of the crude associations between the use of PCPs and IVF/ICSI outcomes were consistent with the adjusted models (**Supplementary Tables 3**, **4**). The association between PCPs characteristics and pregnancy outcomes in fresh cycles revealed that women who used cosmetics 1–2 times per week (adjusted OR = 2.2, 95% CI: 1.0, 4.8) or 3–7 times per week (adjusted OR = 2.5, 95% CI: 1.2, 5.2) had a higher possibility of miscarriage (**Table 4**). When we further restricted the analysis to participants without PCOS (**Supplementary Tables 5–7**), to participants without endometriosis (**Supplementary Tables 8–10**), and to the participants without male–factor infertility, the results were still stable (**Supplementary Tables 11–13**).

**Table 4:** Associations between PCPs characteristics and IVF/ICSI pregnancy outcomes among 947 cycles with fresh embryos transferred.

Characteristics (times per week)	Implantation	Clinical pregnancy	Miscarriage	Live birth
	OR (95% CI)	OR (95% CI)	OR (95% CI)	OR (95% CI)
Use of gel or soap				
0	Ref	Ref	Ref	Ref
1~<3	0.60 (0.31, 1.2)	0.93 (0.49, 1.8)	1.0 (0.29, 4.1)	0.95 (0.50, 1.8)
3~<7	0.63 (0.33, 1.1)	0.97 (0.54, 1.7)	1.2 (0.39, 4.3)	0.97 (0.54, 1.8)
≥7	0.71 (0.38, 1.3)	0.95 (0.54, 1.7)	0.90 (0.31, 3.3)	1.0 (0.57, 1.8)
Use of shampoo				
≤1	Ref	Ref	Ref	Ref
2~≤3	1.2 (0.64, 2.4)	1.3 (0.67, 2.6)	0.88 (0.25, 4.2)	1.3 (0.69, 2.7)
4~<7	1.5 (0.76, 2.8)	1.5 (0.81, 3.0)	0.82 (0.24, 3.8)	1.6 (0.83, 3.2)
≥7	1.2 (0.54, 2.6)	0.84 (0.38, 1.9)	0.96 (0.18, 5.7)	0.89 (0.39, 2.1)
Use of skin care products				
0	Ref	Ref	Ref	Ref
1~<7	0.81 (0.48, 1.4)	0.85 (0.51, 1.4)	1.3 (0.50, 3.7)	0.80 (0.48, 1.34)
7~<14	0.93 (0.58, 1.5)	0.90 (0.57, 1.4)	0.77 (0.31, 2.1)	0.96 (0.61, 1.53)
≥14	0.81 (0.49, 1.3)	0.74 (0.45, 1.2)	1.3 (0.48, 3.6)	0.71 (0.43, 1.17)
Use of cosmetics				
0	Ref	Ref	Ref	Ref
1~≤2	0.92 (0.62, 1.4)	0.94 (0.63, 1.4)	**2.2 (1.0, 4.8)** *	0.76 (0.51, 1.1)
3~<7	0.92 (0.62, 1.4)	0.88 (0.60, 1.3)	**2.5 (1.2, 5.2)** *	0.70 (0.47, 1.0)
≥7	0.90 (0.64, 1.3)	0.91 (0.65, 1.3)	1.0 (0.46, 2.2)	0.91 (0.65, 1.3)
Dyeing or perming hair in past 3 months				
No	Ref	Ref	Ref	Ref
Yes	1.1 (0.78, 1.6)	1.1 (0.86, 1.8)	1.1 (0.75, 2.8)	1.1 (0.75 1.6)

Generalized linear regression models were adjusted for age, BMI, passive smoking, alcohol consumption, education level and income. Bold indicates that the item has statistical significance. *P < 0.05.

PCPs, personal care products; IVF, in vitro fertilization; ICSI, intracytoplasmic sperm injection; OR, odd ratio; Ref, reference.

## 4 Discussion

Based on the TREE cohort, we analyzed the association between PCPs use and the reproductive outcomes of IVF/ICSI treatment. The results indicated that among women undergoing IVF/ICSI treatment, women who used skin care products more frequently (≥14 times per week) had lower oocyte maturation rates than those who did not use skin care products. After the transfer of fresh embryos, cosmetic users (1~2 and 3~<7 times per week) had a higher risk of miscarriage than non-users.

As previously mentioned, limited research exists on the association between PCPs use and reproductive outcomes. A recent study showed that women who use soap during pregnancy had a longer mean birth length and a longer mean gestational age at delivery compared to non-users (33). A case-control study from the United States found null association between perinatal use of chemical curl products and preterm birth (34). Another prospective cohort study involving 9710 pregnant women reported an increased risk of small-for-gestational-age infants among those with a high frequency of cosmetics use (≥5 times per week) during pregnancy (35). In addition, studies on hairdressers revealed that they have an increased risk of spontaneous abortion compared to the normal women (36, 37). Although these studies share some similarities with ours, none have reported on the association between PCPs use and oocyte maturation.

There is increasing evidence that the PCPs use patterns influence the levels of exposure markers to EDCs in human specimens. A Korean study involving 5,962 participants showed that urinary concentrations of methylparaben (MeP), ethylparaben (EtP) and propylparaben (PrP) are directly correlated with the use of PCPs (13). PCPs were also the major source of PAE exposure in women (38), and urinary concentrations of MeP, EtP and PrP were notably higher in females than in males (13, 39). Moreover, in a Taiwan birth cohort of 281 pregnant women, the use patterns of skin care products and cosmetics were positively associated with urinary PAEs concentrations. Notably, leave–on PCPs (e.g., toners, lipsticks, essential oils) had stronger associations than rinse–off PCPs (e.g., shampoo, face wash) (40).

Previous studies have reported the associations between internal exposure levels of single or mixed EDCs and female reproductive function, but these have overlooked the potential correlation between the use of PCPs and female fertility. The current study found that the use of skin care products was negatively associated with oocyte maturation in IVF/ICSI treatments. In prior research, urinary concentrations of phthalates, parabens and glycol ethers in women were associated with time to pregnancy, which represents a quantification of the ability to conceive (41). Moreover, among women undergoing IVF treatment in Poland, the urinary concentration of butylparaben (BuP) was also associated with reduced numbers of MII oocytes but had a null association with the rates of implantation, clinical pregnancy, and live birth (42). Animal studies have also found that several ingredients in sunscreens, such as BPs and nanoparticles (NPs), to diminished oocyte developmental potential. For instance, exposure to UV filters 3-benzylidene camphor or BP-2 resulted in decreased ovulation, fewer mature oocytes, and more atretic follicles in *Pimephales promelas* (43, 44). NPs were shown to cause increased follicular atresia and disrupt follicular maturation in mice (45). ZnO can impair mouse oocyte meiosis and early embryonic development by promoting mitochondrial stress and endoplasmic reticulum stress, activating autophagy and apoptosis (46). TiO2NPs were associated with reduced oocyte number, fertilization rate, cleaved-embryo and blastocyst counts (47). Additionally, components like PPD, ethylene glycol butyl ester, and octocrylene found in hair dyes or cosmetics were associated with mitochondrial dysfunction, leading to impaired oocyte quality (48–50).

Although it is recommended in clinical practice that pregnant women reduce the use of PCPs, Lang et al. (51) found consistent use patterns of hygiene and skincare products throughout pregnancy, whereas the use of cosmetics and hair styling products decreased during this period. Since a variety of BPs and parabens have been found in umbilical cord blood (52) and the concentrations of TCS and TCC in umbilical cord blood are positively correlated with those in maternal serum (53), the fetus may be affected by maternal exposure to PCPs during growth and development. Our study showed that cosmetic use increased the risk of miscarriage. Furthermore, urinary concentrations of bisphenol analogs and phthalates were also reported to be associated with recurrent miscarriage in case–control studies (54–56). A birth cohort from New York reported that butylparaben concentrations in cord blood were associated with an increased risk of preterm birth and decreased gestational age at birth (57). Occupational exposure populations, such as cosmetologists, demonstrated a higher risk of pregnancy complications, like fetal growth restriction, compared to the general population (58, 59). In animal studies, BPA exposure was associated with reduced embryo implantation (60). Benzophenone–3 may induce placental thrombosis and miscarriage (61). Maternal exposure to TiO2NPs was associated with increased atretic follicles, decreased mating and pregnancy rates (62), increased the number of stunted fetuses, and increased fetal mortality (63).

The association between PCPs use and female reproductive function can potentially be explained through several biological mechanisms. Oocyte maturation is susceptible to oxidative stress. For instance, propyl gallate, an ingredient present in cosmetics, may induce oxidative stress and DNA damage, potentially affecting methylation levels and influencing the meiotic maturation of oocytes (64). Moreover, PFOA and PAEs can promote oxidative stress level, inducing apoptosis and necrosis in oocyte (6, 65). During pregnancy, increased oxidative stress, impaired placental function and luteal function, and autoimmune disease may impact embryonic development and even lead to miscarriage. For instance, di-(2–ethylhexyl) phthalate (DEHP) and mono(2–ethylhexyl) phthalate have been reported to affect placental function by altering angiogenesis and promoting oxidative stress (66). In early pregnant mice, exposure to ZnO NP promotes oxidative stress and mitochondrial apoptosis *in utero*, leading to increased rates of miscarriage (67). In addition, prenatal PFOA exposure inhibited ovarian luteal function in mice, leading to increased embryonic resorption (68). In a prospective study involving patients with recurrent miscarriage, serum BPA levels were significantly higher in antinuclear antibody positive patients than in negative patients (69).

In stratified analyses, we observed that the association of PCPs use with IVF/ICSI parameters varied with age and BMI. It has been shown that adult urinary paraben concentrations are negatively associated with age (70) and that younger women tend to use more toiletries (71). Our results align with these observations, particularly noting the negative association between the use of gel or soap and the cleavage rate observed specifically in women under 30 years of age. Moreover, Wenzel et al. (72) found a positive association between BMI and urinary phthalate concentration. Similarly, our study observed that the use of cosmetics was negatively associated with the maturation rate exclusively in women with BMI ≥ 24 kg/m^2^. These findings suggest that there might be interaction effects between age, BMI, and PCPs use in women. However, further research into the underlying mechanisms driving these interactions is necessary for a comprehensive understanding of their implications.

According to our knowledge, this study is the first to examine the associations between habitual PCPs usage and IVF/ICSI outcomes. Our strengths lie in the prospective study design, a substantial sample size, and comprehensive data on PCPs use and clinical information. In addition, the current study investigates the direct use of PCPs within infertile patients, which is closer to daily life, offering valuable implications for women planning pregnancy or undergoing fertility treatments. However, certain limitations need to be acknowledged. Firstly, we used the frequency of PCPs use but not the concentrations of EDCs within PCPs for subsequent analysis. Secondly, the PCPs characteristics gathered from questionnaires represent external exposures, potentially leading to inaccuracies due to individual variations in absorption, metabolism, and exposure pathways. Thirdly, the questionnaire did not include information on brands and dosages of PCPs. Fourthly, the information obtained from the questionnaire in a prospective cohort might not entirely represent the PCPs use patterns during pregnancy, possibly introducing bias into the results. Finally, the participants in the current study were recruited from an infertility cohort undergoing IVF/ICSI treatment, potentially limiting the generalizability of the findings to the broader population. Despite not measuring internal exposure levels, this study offers valuable insights into the association between PCPs use and pregnancy outcomes.

## 5 Conclusions

In conclusion, our results indicate that the use of PCPs in women undergoing IVF/ICSI treatment was associated with a decreased oocyte maturation rate and an increased miscarriage rate. This hints at the potential impact of exposure to a mixture of EDCs found in PCPs on female fertility. The current study provides information on the associations between PCPs and adverse reproductive outcomes, recommending caution regarding PCPs use during pregnancy preparation. However, further epidemiologic studies are needed for further validation.

## Data availability statement

The raw data supporting the conclusions of this article will be made available by the authors, without undue reservation.

## Ethics statement

This study was approved by Ethics Committee of Tongji Hospital, Tongji Medical College, Huazhong University of Science and Technology. This study was conducted in accordance with the local legislation and institutional requirements. The participants provided their written informed consent to participate in this study.

## Author contributions

Q-CG: Conceptualization, Data curation, Formal Analysis, Investigation, Methodology, Writing – original draft, Writing – review & editing. WY: Formal Analysis, Investigation, Writing – review & editing. CL: Methodology, Investigation, Validation. T-RD: Investigation, Writing – review & editing. JL: Investigation, Methodology, Writing – review & editing. H-ML: Investigation, Methodology, Writing – review & editing. W-QT: Investigation, Methodology, Writing – review & editing. YW: Investigation, Methodology, Writing – review & editing. Y-YD: Formal Analysis, Methodology, Supervision, Writing – review & editing. Y-FL: Conceptualization, Funding acquisition, Project administration, Supervision, Writing – review & editing.
