# Supplementary material for: Associations of personal care products use with reproductive outcomes of IVF/ICSI treatment
Source: Front Endocrinol (Lausanne). 2024 Jan 24;14:1320893. doi: 10.3389/fendo.2023.1320893 (PMC10847553; doi:10.3389/fendo.2023.1320893)
Supplement: Supplementary file 1 [file DataSheet_1.docx]

Supplementary Material

Associations of Personal Care Products Use with Reproductive Outcomes of IVF/ICSI Treatment

Qing-Chun Guo, Wen Yao, Chong Liu, Tao-Ran Deng, Juan Li, Hong-Mei Liao, Wen-Qu Tian, Yi Wang, Yao-Yao Du^1^, and Yufeng Li

**Tables of Contents**

**Table S1.** Associations between the use of PCPs (continuous) and IVF/ICSI reproductive outcomes stratified by age (n=1500).

**Table S2.** Associations between the use of PCPs (continuous) and IVF/ICSI reproductive outcomes stratified by BMI (n=1500).

**Table S3.** Crude associations between PCPs characteristics and IVF/ICSI parameters (n=1500).

**Table S4.** Crude associations between PCPs characteristics and IVF/ICSI pregnancy outcomes among 1364 women with embryos transferred in the TREE cohort.

**Table S5.** Associations between PCPs characteristics and IVF/ICSI parameters after excluding participants with PCOS (n=1352).

**Table S6.** Associations between PCPs characteristics and IVF/ICSI pregnancy outcomes among 1224 cycles with embryos transferred after excluding participants with PCOS.

**Table S7.** Associations between PCPs characteristics and IVF/ICSI pregnancy outcomes among 868 cycles with fresh embryos transferred after excluding participants with PCOS.

**Table S8.** Associations between PCPs characteristics and IVF/ICSI parameters after excluding participants with endometriosis (n=1422).

**Table S9.** Associations between PCPs characteristics and IVF/ICSI pregnancy outcomes among 1293 cycles with embryos transferred after excluding participants with endometriosis.

**Table S10.** Associations between PCPs characteristics and IVF/ICSI pregnancy outcomes among 895 cycles with fresh embryos transferred after excluding participants with endometriosis.

**Table S11.** Associations between PCPs characteristics and IVF/ICSI parameters after excluding participants with male-factor infertility (n=1333).

**Table S12.** Associations between PCPs characteristics and IVF/ICSI pregnancy outcomes among 1210 cycles with embryos transferred after excluding participants with male-factor infertility.

**Table S13.** Associations between PCPs characteristics and IVF/ICSI pregnancy outcomes among 827 cycles with fresh embryos transferred after excluding participants with male-factor infertility.

**Figure Legends**

**Figure S1.** Directed acyclic graph of intermediate IVF/ICSI outcomes.

**Figure S2.** Directed acyclic graph of early pregnancy outcomes.

| **Supplementary** **Table S1.** Associations between the use of PCPs (continuous) and IVF/ICSI reproductive outcomes stratified by age (n=1500). | | | | | |
| --- | --- | --- | --- | --- | --- |
|  | ≤30 (n=791) | | >30 (n=709) | |  |
|  | *β* (95% CI) | *P* | *β* (95% CI) | *P* | *P* for interaction |
| **Use of gel or soap** |  |  |  |  |  |
| Number of retrieved oocytes | –0.001 (–0.02, 0.02) | 0.94 | –0.01 (–0.03, 0.01) | 0.47 | 0.69 |
| Number of mature oocytes | 0.003 (–0.01, 0.02) | 0.73 | –0.01 (–0.03, 0.01) | 0.24 | 0.33 |
| Maturation rate | 0.01 (–0.02, 0.04) | 0.55 | –0.02 (–0.06, 0.02) | 0.37 | 0.34 |
| Number of 2PN zygotes | 0.01 (–0.01, 0.02) | 0.56 | –0.01 (–0.03, 0.01) | 0.33 | 0.36 |
| Fertilization rate | 0.01 (–0.02, 0.04) | 0.58 | 0.02 (–0.01, 0.05) | 0.25 | 0.57 |
| Cleavage rate | –0.16 (–0.27, –0.06) | **0.004** | 0.07 (–0.05, 0.20) | 0.23 | **0.01** |
| Blastocyst formation rate | –0.04 (–0.08, 0.002) | 0.07 | 0.02 (–0.03, 0.07) | 0.39 | **0.04** |
| **Use of shampoo** |  |  |  |  |  |
| Number of retrieved oocytes | –0.01 (–0.04, 0.02) | 0.45 | 0.01 (–0.02, 0.05) | 0.38 | 0.27 |
| Number of mature oocytes | –0.01 (–0.04, 0.02) | 0.45 | 0.01 (–0.02, 0.05) | 0.52 | 0.34 |
| Maturation rate | –0.01 (–0.07, 0.04) | 0.64 | 0.0004 (–0.07, 0.07) | 0.99 | 0.66 |
| Number of 2PN zygotes | –0.02 (–0.05, 0.01) | 0.25 | 0.02 (–0.02, 0.05) | 0.38 | 0.15 |
| Fertilization rate | –0.001 (–0.05, 0.05) | 0.98 | 0.03 (–0.03, 0.09) | 0.31 | 0.36 |
| Cleavage rate | –0.04 (–0.20, 0.13) | 0.61 | 0.02 (–0.19, 0.27) | 0.85 | 0.62 |
| Blastocyst formation rate | 0.05 (–0.02, 0.12) | 0.17 | 0.01 (–0.07, 0.09 | 0.81 | 0.51 |
| **Use of skin care products** |  |  |  |  |  |
| Number of retrieved oocytes | –0.002 (–0.01, 0.01) | 0.70 | 0.01 (0.003, 0.02) | **0.01** | **0.02** |
| Number of mature oocytes | –0.001 (–0.01, 0.01) | 0.78 | 0.01 (0.002, 0.02) | **0.02** | **0.04** |
| Maturation rate | –0.003 (–0.02, 0.02) | 0.78 | –0.02 (–0.04, –0.001) | **0.04** | 0.11 |
| Number of 2PN zygotes | –0.002 (–0.01, 0.01) | 0.66 | 0.01 (0.0003, 0.02) | **0.04** | 0.07 |
| Fertilization rate | –0.003 (–0.02, 0.01) | 0.71 | 0.01 (–0.01, 0.03) | 0.38 | 0.36 |
| Cleavage rate | –0.03 (–0.08, 0.03) | 0.36 | 0.01 (–0.06, 0.08) | 0.74 | 0.448 |
| Blastocyst formation rate | 0.01 (–0.01, 0.03) | 0.33 | 0.02 (–0.004, 0.05) | 0.10 | 0.56 |
| **Use of cosmetics** |  |  |  |  |  |
| Number of retrieved oocytes | 0.002 (–0.01, 0.01) | 0.75 | 0.001 (–0.01, 0.01) | 0.87 | 0.98 |
| Number of mature oocytes | 0.004 (–0.01, 0.02) | 0.49 | –0.0002 (–0.01, 0.01) | 0.96 | 0.66 |
| Maturation rate | 0.02 (–0.01, 0.05) | 0.20 | –0.01 (–0.02, 0.01) | 0.47 | 0.16 |
| Number of 2PN zygotes | 0.06 (–0.01, 0.02) | 0.44 | 0.001 (–0.01, 0.01) | 0.80 | 0.75 |
| Fertilization rate | 0.0001 (–0.02, 0.02) | 0.99 | 0.01 (–0.01, 0.03) | 0.47 | 0.55 |
| Cleavage rate | –0.06 (–0.13, 0.02) | 0.13 | 0.01 (–0.04, 0.11) | 0.78 | 0.22 |
| Blastocyst formation rate | 0.01 (–0.02, 0.04) | 0.47 | 0.002 (–0.02, 0.03) | 0.83 | 0.79 |

Note: Generalized linear regression models were adjusted for age, BMI, passive smoking, alcohol consumption, education level, and income. Bold indicates that the item has statistical significance.

Abbreviations: PCPs, personal care products; IVF, *in vitro* fertilization; ICSI, intracytoplasmic sperm injection; 2PN, two distinct pronuclei; BMI, body mass index.

| **Supplementary** **Table S2.** Associations between the use of PCPs (continuous) and IVF/ICSI reproductive outcomes stratified by BMI (n=1500). | | | | | |
| --- | --- | --- | --- | --- | --- |
|  | <24 kg/m^2^ (n=1120) | | ≥24 kg/m^2^ (n=378) | |  |
|  | *β* (95% CI) | *P* | *β* (95% CI) | *P* | *P* for interaction |
| **Use of gel or soap** |  |  |  |  |  |
| Number of retrieved oocytes | –0.01 (–0.02, 0.01) | 0.34 | –0.01 (–0.03, 0.02) | 0.67 | 0.85 |
| Number of mature oocytes | –0.01 (–0.02, 0.01) | 0.39 | –0.01 (–0.03, 0.01) | 0.53 | 0.92 |
| Maturation rate | .01 (–0.02, 0.03) | 0.73 | –0.04 (–0.10, 0.02) | 0.16 | 0.12 |
| Number of 2PN zygotes | –0.003 (–0.02, 0.01) | 0.72 | –0.01 (–0.04, 0.02) | 0.47 | 0.61 |
| Fertilization rate | 0.01 (–0.01, 0.04) | 0.28 | 0.02 (–0.03, 0.06) | 0.47 | 0.94 |
| Cleavage rate | –0.04 (–0.13, 0.05) | 0.40 | –0.07 (–0.24, 0.09) | 0.39 | 0.77 |
| Blastocyst formation rate | 0.002 (–0.03, 0.04) | 0.92 | –0.08 (–0.14, –0.01) | **0.02** | **0.04** |
| **Use of shampoo** |  |  |  |  |  |
| Number of retrieved oocytes | –0.01 (–0.03, 0.02) | 0.65 | –0.02 (–0.05, 0.04) | 0.92 | 0.83 |
| Number of mature oocytes | –0.003 (–0.03, 0.02) | 0.79 | –0.02 (–0.06, 0.03) | 0.48 | 0.63 |
| Maturation rate | 0.02 (–0.04, 0.07) | 0.55 | –0.07 (–0.16, 0.02) | 0.13 | 0.07 |
| Number of 2PN zygotes | –0.01 (–0.04, 0.02) | 0.46 | 0.001 (–0.05, 0.05) | 0.98 | 0.78 |
| Fertilization rate | 0.01 (–0.03, 0.06) | 0.55 | 0.01 (–0.07, 0.10) | 0.76 | 0.87 |
| Cleavage rate | –0.08 (–0.21, 0.07) | 0.27 | 0.36 (0.03, 075) | 0.051 | **0.047** |
| Blastocyst formation rate | 0.03 (–0.03, 0.09) | 0.37 | 0.03 (–0.08, 0.15) | 0.59 | 0.98 |
| **Use of skin care products** |  |  |  |  |  |
| Number of retrieved oocytes | 0.002 (–0.01, 0.01) | 0.58 | 0.01 (–0.001, 0.02) | 0.07 | 0.22 |
| Number of mature oocytes | 0.001 (–0.01, 0.01) | 0.79 | 0.01 (0.001, 0.03) | **0.03** | 0.12 |
| Maturation rate | –0.02 (–0.03, 0.001) | 0.07 | –0.004 (–0.03, 0.02) | 0.80 | 0.50 |
| Number of 2PN zygotes | 0.0002 (–0.01, 0.01) | 0.95 | 0.01 (–0.003, 0.03) | 0.10 | 0.24 |
| Fertilization rate | 0.003 (–0.01, 0.02) | 0.64 | –0.004 (–0.03, 0.02) | 0.77 | 0.48 |
| Cleavage rate | –0.01 (–0.06, 0.04) | 0.74 | –0.02 (–0.10, 0.06) | 0.67 | 0.98 |
| Blastocyst formation rate | 0.03 (0.01, 0.05) | **0.01** | –0.02 (–0.05, 0.04) | 0.28 | **0.04** |
| **Use of cosmetics** |  |  |  |  |  |
| Number of retrieved oocytes | 0.003 (–0.01, 0.01) | 0.47 | 0.002 (–0.02, 0.02) | 0.85 | 0.95 |
| Number of mature oocytes | 0.004 (–0.004, 0.01) | 0.27 | –0.006(–0.03, 0.01) | 0.53 | 0.38 |
| Maturation rate | 0.01 (–0.004, 0.04) | 0.16 | –0.05 (–0.09, –0.02) | **0.001** | **0.001** |
| Number of 2PN zygotes | 0.01 (–0.003, 0.01) | 0.21 | –0.01 (–0.03, 0.01) | 0.61 | 0.35 |
| Fertilization rate | 0.004 (–0.01, 0.02) | 0.58 | 0.01 (–0.02, 0.04) | 0.63 | 0.93 |
| Cleavage rate | –0.01 (–0.02, 0.01) | 0.34 | –0.01 (–0.03, 0.02) | 0.67 | 0.85 |
| Blastocyst formation rate | –0.01 (–0.02, 0.01) | 0.39 | –0.01 (–0.03, 0.01) | 0.53 | 0.92 |

Note: Generalized linear regression models were adjusted for age, BMI, passive smoking, alcohol consumption, education level and income. Bold indicates that the item has statistical significance.

Abbreviations: PCPs, personal care products; IVF, *in vitro* fertilization; ICSI, intracytoplasmic sperm injection; 2PN, two distinct pronuclei; BMI, body mass index.

| **Supplementary** **Table S3.** Crude associations between PCPs characteristics and IVF/ICSI parameters (n=1500). | | | | | | | |
| --- | --- | --- | --- | --- | --- | --- | --- |
| **Characteristics**  (times per week) | Number of retrieved oocytes | Number of mature oocytes | Maturation rate | Number of  2PN zygotes | Fertilization rate | Cleavage rate | Blastocyst formation rate |
|  | % Change (95% CI) | % Change (95% CI) | % Change (95% CI) | % Change (95% CI) | % Change (95% CI) | % Change (95% CI) | % Change (95% CI) |
| **Use of gel or soap** | |  |  |  |  |  |  |
| 0 | Ref | Ref | Ref | Ref | Ref | Ref | Ref |
| 1~<3 | –5.6 (–18.5, 9.6) | –8.6 (–21.5, 6.6) | –8.4 (–33.5, 25.0) | –8.6 (–22.8, 8.6) | 1.5 (–21.7, 31.2) | 21.9 (–53.2, 195.3) | 18.3 (–16.5, 67.4) |
| 3~<7 | 3.7 (–9.0, 18.7) | –0.39 (–13.0, 14.4) | –17.7 (–38.6, 8.9) | 1.3 (–12.8, 18.3) | 11.0 (–12.4, 40.2) | 46.5 (–40.5, 222.0) | 29.0 (–6.2, 76.6) |
| ≥7 | 0.61 (–11.3, 14.6) | –2.6 (–14.4, 11.3) | –12.8 (–34.3, 14.2) | –0.99 (–14.2, 15.0) | 13.6 (–9.5, 42.0) | –5.1 (–59.5, 91.8) | 6.2 (–21.7, 43.4) |
| **Use of shampoo** | |  |  |  |  |  |  |
| ≤1 | Ref | Ref | Ref | Ref | Ref | Ref | Ref |
| 2~≤3 | 12.5 (–3.1, 31.4) | 8.2 (–7.2, 27.0) | –10.6 (–35.3, 21.3) | 7.1 (–9.6, 27.9) | –0.04 (–23.6, 29.8) | 53.8 (–33.8, 217.5) | 26.7 (–10.3, 78.4) |
| 4~<7 | 10.5 (–4.5, 28.7) | 6.9 (–8.0, 25.0) | –9.2 (–33.8, 22.3) | 5.1 (–10.9, 25.0) | 6.3 (–18.2, 37.1) | 70.6 (–25.2, 241.9) | 37.3 (–2.0, 91.6) |
| ≥7 | 7.3 (–10.7, 29.1) | 3.6 (–14.2, 25.5) | –6.3 (–36.1, 36.4) | 0.40 (–18.6, 24.1) | 1.5 (–26.4, 39.7) | 8.3 (–58.3, 169.9) | 37.4 (–9.6, 109.1) |
| **Use of skin care products** | |  |  |  |  |  |  |
| 0 | Ref | Ref | Ref | Ref | Ref | Ref | Ref |
| 1~<7 | **16.0 (2.9, 30.9)** * | 13.3 (0.02, 28.4) | **–22.3 (–39.4, –0.93)** * | **18.6 (3.3, 36.4)** * | 13.0 (–8.0, 38.8) | –17.7 (–64.9, 80.4) | 15.0 (–13.3, 52.5) |
| 7~<14 | **14.4 (3.0, 27.4)** * | **13.6 (1.8, 27.0)** * | –9.3 (–27.5, 12.7) | **17.1 (3.7, 32.7)** * | 13.1 (–5.5, 35.1) | –35.3 (–70.1, 24.6) | 16.2 (–9.4, 48.8) |
| ≥14 | **17.4 (4.8, 31.7)** ** | **15.6 (2.8, 30.3)** * | **–22.7 (–39.0, –2.6)** * | **17.6 (3.1, 34.4)** * | 10.9 (–8.8, 34.5) | –21.3 (–65.3, 63.3) | 29.7 (–0.9, 69.7) |
| **Use of cosmetics** | |  |  |  |  |  |  |
| 0 | Ref | Ref | Ref | Ref | Ref | Ref | Ref |
| 1~≤2 | **13.1 (3.2, 23.8)** ** | **12.6 (2.3, 23.8)** * | 0.71 (–16.9, 22.6) | 9.4 (–1.7, 21.6) | –7.2 (–21.3, 9.6) | –6.1 (–45.7, 70.6) | 17.7 (–6.0, 47.7) |
| 3~<7 | 4.8 (–4.0, 14.4) | 5.3 (–3.9, 15.3) | 5.3 (–12.3, 26.8) | 6.9 (–3.4, 18.1) | 11.6 (–4.7, 31.0) | 62.1 (–12.4, 225.9) | –1.4 (–19.9, 21.5) |
| ≥7 | 4.9 (–2.9, 13.3) | 5.5 (–2.7, 14.3) | 3.5 (–11.9, 21.7) | 6.4 (–2.7, 16.2) | 5.1 (–8.4, 20.8) | –19.9 (–48.8, 26.8) | 15.2 (–4.6, 39.4) |
| **Dyeing or perming hair in past 3 months** | | |  |  |  |  |  |
| No | Ref | Ref | Ref | Ref | Ref | Ref | Ref |
| Yes | –2.4 (–10.3, 6.0) | –4.2 (–12.3, 4.5) | –4.6 (–19.3, 13.3) | –2.4 (–11.5, 7.3) | –1.8 (–15.3, 14.0) | –14.8 (–47.0, 43.8) | 6.7 (–12.9, 30.9) |

Note: Generalized linear regression models without covariates. Bold indicates that the item has statistical significance. **P* < 0.05, ***P* < 0.01.

Abbreviations: PCPs, personal care products; IVF, *in vitro* fertilization; ICSI, intracytoplasmic sperm injection; Ref, reference.

| **Supplementary** **Table S4.** Crude associations between PCPs characteristics and IVF/ICSI pregnancy outcomes among 1364 women with embryos transferred in the TREE cohort. | | | | |
| --- | --- | --- | --- | --- |
| **Characteristics**  (times per week) | Implantation | Clinical pregnancy | Miscarriage | Live birth |
|  | OR (95% CI) | OR (95% CI) | OR (95% CI) | OR (95% CI) |
| **Use of gel or soap** | |  |  |  |
| 0 | Ref | Ref | Ref | Ref |
| 1~<3 | 1.1 (0.68, 1.9) | 1.2 (0.72, 2.0) | 1.2 (0.47, 3.6) | 1.1 (0.68, 1.8) |
| 3~<7 | 1.4 (0.90, 2.3) | **1.6 (1.0, 2.5)** * | 0.86 (0.36, 2.4) | **1.6 (1.0, 2.4)** * |
| ≥7 | 1.2 (0.77, 1.9) | 1.2 (0.80, 1.9) | 0.62 (0.26, 1.7) | 1.3 (0.86, 2.0) |
| **Use of shampoo** | |  |  |  |
| ≤1 | Ref | Ref | Ref | Ref |
| 2~≤3 | 1.4 (0.87, 2.3) | 1.3 (0.82, 2.2) | 0.45 (0.19, 1.2) | 1.6 (0.96, 2.6) |
| 4~<7 | 1.5 (0.93, 2.4) | 1.5 (0.90, 2.3) | 0.45 (0.20, 1.2) | **1.7 (1.1, 2.7)** * |
| ≥7 | 1.4 (0.79, 2.7) | 1.2 (0.67, 2.2) | 0.51 (0.15, 1.6) | 1.4 (0.78, 2.5) |
| **Use of skin care products** | |  |  |  |
| 0 | Ref | Ref | Ref | Ref |
| 1~<7 | 1.4 (0.75, 2.7) | 1.3 (0.85, 1.9) | 0.55 (0.24, 1.2) | 1.4 (0.95, 2.1) |
| 7~<14 | **1.5 (1.0, 2.1)** * | **1.4 (1.0, 2.0)** * | 0.55 (0.28, 1.1) | **1.6 (1.1, 2.2)** * |
| ≥14 | 1.4 (0.95, 2.0) | 1.4 (1.0, 2.0) | 0.65 (0.32, 1.4) | **1.5 (1.1, 2.2)** * |
| **Use of cosmetics** | |  |  |  |
| 0 | Ref | Ref | Ref | Ref |
| 1~≤2 | 1.2 (0.85, 1.7) | 1.2 (0.86, 1.7) | 0.68 (0.30, 1.4) | 1.3 (0.91, 1.7) |
| 3~<7 | 1.1 (0.77, 1.4) | 1.2 (0.85, 1.6) | 1.3 (0.70, 2.3) | 1.1 (0.79, 1.4) |
| ≥7 | 1.1 (0.80, 1.4) | 1.1 (0.86, 1.5) | 0.81 (0.44, 1.4) | 1.2 (0.89, 1.5) |
| **Dyeing or perming hair in past 3 months** | | |  |  |
| No | Ref | Ref | Ref | Ref |
| Yes | 1.1 (0.70, 1.3) | 1.1 (0.75, 1.3) | 1.1 (0.62, 2.0) | 1.1 (0.74, 1.3) |

Note: Generalized linear regression models without covariates. Bold indicates that the item has statistical significance. **P* < 0.05.

Abbreviations: PCPs, personal care products; IVF, *in vitro* fertilization; ICSI, intracytoplasmic sperm injection; OR, odd ratio; Ref, reference.

| **Supplementary Table S5.** Associations between PCPs characteristics and IVF/ICSI parameters after excluding participants with PCOS (n=1352). | | | | | | | |
| --- | --- | --- | --- | --- | --- | --- | --- |
| **Characteristics**  (times per week) | Number of retrieved oocytes | Number of mature oocytes | Maturation rate | Number of  2PN zygotes | Fertilization rate | Cleavage rate | Blastocyst formation rate |
|  | % Change (95% CI) | % Change (95% CI) | % Change (95% CI) | % Change (95% CI) | % Change (95% CI) | % Change (95% CI) | % Change (95% CI) |
| **Use of gel or soap** | |  |  |  |  |  |  |
| 0 | Ref | Ref | Ref | Ref | Ref | Ref | Ref |
| 1~<3 | –1.7 (–15.2, 14.2) | –5.3 (–18.8, 10.6) | –7.8 (–35.1, 29.6) | –5.6 (–20.7, 12.7) | 0.44 (–24.1, 32.4) | 30.5 (–51.6, 226.9) | 18.9 (–18.7, 73.5) |
| 3~<7 | –1.6 (–13.8, 12.7) | –5.6 (–17.7, 8.6) | –15.3 (–38.6, 15.0) | –3.8 (–17.6, 13.0) | 11.7 (–13.6, 43.8) | 75.9 (–31.4, 303.2) | 28.1 (–9.5, 80.7) |
| ≥7 | –4.3 (–15.7, 9.1) | –8.0 (–19.3, 5.4) | –10.7 (–34.6, 19.9) | –5.4 (–18.5, 10.5) | 15.4 (–9.9, 47.0) | 10.6 (–54.3, 130.3) | 2.2 (–26.8, 42.1) |
| **Use of shampoo** | |  |  |  |  |  |  |
| ≤1 | Ref | Ref | Ref | Ref | Ref | Ref | Ref |
| 2~≤3 | 2.4 (–11.4, 19.1) | –2.1 (–15.7, 14.4) | –14.3 (–40.0, 19.8) | –4.5 (–19.3, 13.8) | –7.7 (–30.7, 22.1) | 15.6 (–60.6, 177.3) | 14.2 (–21.2, 64.6) |
| 4~<7 | 2.4 (–11.4, 19.1) | –6.3 (–19.0, 9.2) | –13.4 (–38.9, 20.1) | –7.5 (–21.5, 9.8) | 3.0 (–22.2, 35.4) | 23.1 (–57.3, 186.0) | 16.7 (–18.7, 66.8) |
| ≥7 | 2.8 (–13.8, 23.0) | –2.2 (–18.7, 17.7) | –14.2 (–43.5, 29.0) | –4.8 (–22.6, 17.4) | –0.98 (–29.7, 39.1) | –25.3 (–76.9, 113.1) | 17.1 (–25.1, 82.8) |
| **Use of skin care products** | |  |  |  |  |  |  |
| 0 | Ref | Ref | Ref | Ref | Ref | Ref | Ref |
| 1~<7 | 8.0 (–4.4, 22.2) | 6.0 (–6.7, 20.6) | –19.7 (–39.0, 5.2) | 9.5 (–5.2, 26.7) | 8.5 (–13.5, 35.8) | –48.8 (–83.3, 32.9) | –0.59 (–27.4, 35.9) |
| 7~<14 | 8.6 (–2.6, 21.4) | 8.5 (–3.2, 21.9) | –7.8 (–28.4, 17.7) | 9.1 (–4.1, 24.6) | 7.6 (–11.9, 31.2) | **–61.0 (–86.5, –9.8)** * | 6.6 (–19.6, 40.9) |
| ≥14 | 10.7 (–1.5, 24.6) | 10.0 (–2.6, 24.6) | –22.0 (–40.2, 1.1) | 11.4 (–3.0, 28.3) | 10.3 (–11.2, 36.8) | –53.8 (–84.5, 13.8) | 15.6 (–14.5, 56.1) |
| **Use of cosmetics** | |  |  |  |  |  |  |
| 0 | Ref | Ref | Ref | Ref | Ref | Ref | Ref |
| 1~≤2 | 6.9 (–2.4, 16.9) | 6.4 (–3.3, 16.9) | 3.0 (–16.4, 27.5) | 3.8 (–6.9, 15.5) | –4.1 (–19.7, 14.8) | –18.3 (–54.9, 54.8) | 7.5 (–15.5, 37.2) |
| 3~<7 | 0.05 (–8.4, 9.1) | 0.54 (–8.3, 10.2) | 8.0 (–11.5, 32.3) | 1.5 (–8.5, 12.4) | 10.1 (–7.0, 30.6) | 44.8 (–25.7, 207.4) | –11.0 (–28.9, 11.5) |
| ≥7 | 4.1 (–3.8, 12.6) | 6.4 (–3.3, 16.9) | 2.2 (–14.5, 22.3) | 4.7 (–4.7, 14.9) | 4.9 (–9.8, 22.2) | –29.4 (–57.3, 18.1) | 0.33 (–18.5, 23.6) |
| **Dyeing or perming hair in past 3 months** | | |  |  |  |  |  |
| No | Ref | Ref | Ref | Ref | Ref | Ref | Ref |
| Yes | 3.3 (–4.8, 12.0) | 0.48 (–7.9, 9.5) | –9.1 (–24.0, 9.2) | 3.2 (–6.4, 13.5) | 0.02 (–14.5, 17.2) | –23.0 (–53.4, 33.8) | 4.3 (–15.8, 29.5) |

Note: Generalized linear regression models were adjusted for age, BMI, passive smoking, alcohol consumption, education level and income. Bold indicates that the item has statistical significance. **P* < 0.05.

Abbreviations: PCPs, personal care products; IVF, *in vitro* fertilization; ICSI, intracytoplasmic sperm injection; PCOS, polycystic ovary syndrome; 2PN, two distinct pronuclei; BMI, body mass index; Ref, reference.

| **Supplementary** **Table S6.** Associations between PCPs characteristics and IVF/ICSI pregnancy outcomes among 1224 cycles with embryos transferred after excluding participants with PCOS. | | | | |
| --- | --- | --- | --- | --- |
| **Characteristics**  (times per week) | Implantation | Clinical pregnancy | Miscarriage | Live birth |
|  | OR (95% CI) | OR (95% CI) | OR (95% CI) | OR (95% CI) |
| **Use of gel or soap** | |  |  |  |
| 0 | Ref | Ref | Ref | Ref |
| 1~<3 | 1.3 (0.71, 2.2) | 1.4 (0.78, 2.4) | 1.5 (0.51, 5.7) | 1.2 (0.69, 2.1) |
| 3~<7 | 1.2 (0.72, 2.1) | 1.5 (0.88, 2.5) | 1.1 (0.40, 4.0) | 1.4 (0.82, 2.3) |
| ≥7 | 1.1 (0.65, 1.8) | 1.1 (0.69, 1.9) | 0.75 (0.27, 2.7) | 1.2 (0.73, 1.9) |
| **Use of shampoo** | |  |  |  |
| ≤1 | Ref | Ref | Ref | Ref |
| 2~≤3 | 1.1 (0.63, 2.0) | 1.0 (0.58, 1.8) | 0.78 (0.27, 2.8) | 1.1 (0.62, 1.9) |
| 4~<7 | 1.1 (0.62, 1.9) | 1.0 (0.60, 1.8) | 0.83 (0.30, 3.0) | 1.1 (0.63, 1.9) |
| ≥7 | 1.2 (0.58, 2.3) | 0.97 (0.49, 1.9) | 1.1 (0.28, 4.5) | 0.97 (0.50, 1.9) |
| **Use of skin care products** | |  |  |  |
| 0 | Ref | Ref | Ref | Ref |
| 1~<7 | 0.87 (0.54, 1.4) | 1.0 (0.63, 1.6) | 0.89 (0.34, 2.4) | 1.0 (0.67, 1.6) |
| 7~<14 | 1.2 (0.77, 1.8) | 1.2 (0.77, 1.7) | 0.89 (0.39, 2.2) | 1.2 (0.79, 1.8) |
| ≥14 | 1.1 (0.68, 1.7) | 1.1 (0.73, 1.7) | 1.0 (0.43, 2.7) | 1.1 (0.73, 1.7) |
| **Use of cosmetics** | |  |  |  |
| 0 | Ref | Ref | Ref | Ref |
| 1~≤2 | 0.92 (0.63, 1.3) | 0.95 (0.66, 1.36) | 0.93 (0.38, 2.1) | 0.97 (0.68, 1.4) |
| 3~<7 | 0.93 (0.66, 1.3) | 1.0 (0.75, 1.48) | 1.9 (0.95, 3.5) | 0.90 (0.65, 1.3) |
| ≥7 | 1.1 (0.68, 1.4) | 1.1 (0.84, 1.6) | 1.0 (0.51, 2.0) | 1.1 (0.83, 1.5) |
| **Dyeing or perming hair in past 3 months** | | |  |  |
| No | Ref | Ref | Ref | Ref |
| Yes | 1.1 (0.75, 1.4) | 1.1 (0.86, 1.6) | 1.1 (0.53, 2.0) | 1.1 (0.84, 1.6) |

Note: Generalized linear regression models were adjusted for age, BMI, passive smoking, alcohol consumption, education level and income.

Abbreviations: PCPs, personal care products; IVF, *in vitro* fertilization; ICSI, intracytoplasmic sperm injection; PCOS, polycystic ovary syndrome; OR, odd ratio; BMI, body mass index; Ref, reference.

| **Supplementary** **Table S7.** Associations between PCPs characteristics and IVF/ICSI pregnancy outcomes among 868 cycles with fresh embryos transferred after excluding participants with PCOS. | | | | |
| --- | --- | --- | --- | --- |
| **Characteristics**  (times per week) | Implantation | Clinical pregnancy | Miscarriage | Live birth |
|  | OR (95% CI) | OR (95% CI) | OR (95% CI) | OR (95% CI) |
| **Use of gel or soap** | |  |  |  |
| 0 | Ref | Ref | Ref | Ref |
| 1~<3 | 0.68 (0.34, 1.3) | 0.93 (0.48, 1.8) | 0.97 (0.27, 4.0) | 0.97 (0.50, 1.9) |
| 3~<7 | 0.66 (0.34, 1.2) | 0.92 (0.49, 1.7) | 0.98 (0.32, 3.8) | 0.97 (0.52, 1.8) |
| ≥7 | 0.75 (0.40, 1.4) | 0.93 (0.51, 1.7) | 0.69 (0.23, 2.6) | 1.0 (0.57, 1.9) |
| **Use of shampoo** | |  |  |  |
| ≤1 | Ref | Ref | Ref | Ref |
| 2~≤3 | 1.3 (0.64, 2.5) | 1.3 (0.68, 2.7) | 1.5 (0.35, 10.0) | 1.3 (0.64, 2.6) |
| 4~<7 | 1.4 (0.73, 2.8) | 1.6 (0.80, 3.1) | 1.2 (0.30, 8.4) | 1.5 (0.77, 3.1) |
| ≥7 | 0.96 (0.42, 2.2) | 0.80 (0.35, 1.9) | 2.0 (0.32, 17.0) | 0.74 (0.31, 1.8) |
| **Use of skin care products** | |  |  |  |
| 0 | Ref | Ref | Ref | Ref |
| 1~<7 | 0.86 (0.49, 1.5) | 0.91 (0.53, 1.6) | 1.8 (0.58, 6.9) | 0.79 (0.46, 1.4) |
| 7~<14 | 0.99 (0.60, 1.6) | 0.99 (0.61, 1.6) | 1.2 (0.40, 4.3) | 0.96 (0.58, 1.6) |
| ≥14 | 0.82 (0.48, 1.4) | 0.77 (0.45, 1.3) | 2.0 (0.64, 7.5) | 0.67 (0.39, 1.1) |
| **Use of cosmetics** | |  |  |  |
| 0 | Ref | Ref | Ref | Ref |
| 1~≤2 | 0.90 (0.60, 1.4) | 0.92 (0.61, 1.4) | **2.7 (1.2, 6.2)** * | 0.72 (0.47, 1.1) |
| 3~<7 | 0.93 (0.63, 1.4) | 0.91 (0.61, 1.3) | **2.6 (1.1, 5.9)** * | 0.73 (0.49, 1.1) |
| ≥7 | 0.93 (0.65, 1.3) | 0.99 (0.70, 1.4) | 1.2 (0.50, 2.7) | 0.95 (0.67, 1.4) |
| **Dyeing or perming hair in past 3 months** | | |  |  |
| No | Ref | Ref | Ref | Ref |
| Yes | 1.1 (0.79, 1.7) | 1.1 (0.89, 1.9) | 1.1 (0.76, 3.1) | 1.1 (0.78, 1.6) |

Note: Generalized linear regression models were adjusted for age, BMI, passive smoking, alcohol consumption, education level and income. Bold indicates that the item has statistical significance. **P* < 0.05.

Abbreviations: PCPs, personal care products; IVF, *in vitro* fertilization; ICSI, intracytoplasmic sperm injection; PCOS, polycystic ovary syndrome; OR, odd ratio; BMI, body mass index; Ref, reference.

| **Supplementary Table S8.** Associations between PCPs characteristics and IVF/ICSI parameters after excluding participants with endometriosis (n=1422). | | | | | | | |
| --- | --- | --- | --- | --- | --- | --- | --- |
| **Characteristics**  (times per week) | Number of retrieved oocytes | Number of mature oocytes | Maturation rate | Number of  2PN zygotes | Fertilization rate | Cleavage rate | Blastocyst formation rate |
|  | % Change (95% CI) | % Change (95% CI) | % Change (95% CI) | % Change (95% CI) | % Change (95% CI) | % Change (95% CI) | % Change (95% CI) |
| **Use of gel or soap** | |  |  |  |  |  |  |
| 0 | Ref | Ref | Ref | Ref | Ref | Ref | Ref |
| 1~<3 | –3.1 (–15.8, 11.9) | –6.6 (–19.4, 8.6) | –11.3 (–36.2, 22.2) | –6.5 (–20.8, 10.8) | 1.0 (–22.4, 31.1) | 28.0 (–53.5, 226.5) | 20.4 (–15.8, 71.8) |
| 3~<7 | –1.5 (–13.2, 12.2) | –4.9 (–16.7, 8.9) | –17.4 (–38.9, 10.2) | –2.6 (–16.0, 13.4) | 11.8 (–12.2, 41.8) | 46.8 (–43.0, 233.9) | 26.5 (–8.7, 74.7) |
| ≥7 | –3.6 (–14.6, 9.3) | –6.1 (–17.3, 7.0) | –10.9 (–33.5, 17.6) | –4.1 (–16.8, 11.1) | 14.6 (–9.1, 43.9) | –2.8 (–60.4, 103.1) | 4.0 (–24.0, 41.6) |
| **Use of shampoo** | |  |  |  |  |  |  |
| ≤1 | Ref | Ref | Ref | Ref | Ref | Ref | Ref |
| 2~≤3 | 5.6 (–8.5, 22.6) | 1.5 (–12.6, 18.5) | –11.6 (–36.8, 21.4) | 1.2 (–14.4, 20.4) | 1.2 (–23.0, 32.2) | 54.7 (–37.7, 236.3) | 29.2 (–9.4, 83.6) |
| 4~<7 | 1.5 (–11.8, 17.4) | –1.8 (–15.1, 14.4) | –10.0 (–35.2, 22.7) | –3.0 (–17.5, 15.0) | 5.7 (–19.2, 37.2) | 71.8 (–29.6, 262.9) | 35.7 (–4.0, 91.3) |
| ≥7 | 2.6 (–14.1, 22.8) | –0.74 (–17.6, 19.8) | –5.7 (–37.0, 40.3) | –3.8 (–21.9, 18.7) | –3.1 (–30.4, 34.6) | 6.0 (–62.3, 185.8) | 36.8 (–11.4, 111.5) |
| **Use of skin care products** | |  |  |  |  |  |  |
| 0 | Ref | Ref | Ref | Ref | Ref | Ref | Ref |
| 1~<7 | 7.7 (–4.0, 21.0) | 4.3 (–7.6, 17.9) | **–25.6 (–42.5, –4.1)** * | 8.2 (–5.5, 24.1) | 4.9 (–15.1, 29.6) | –22.3 (–67.9, 75.5) | 4.8 (–21.5, 39.7) |
| 7~<14 | 7.5 (–3.0, 19.4) | 6.1 (–4.8, 18.5) | –12.5 (–31.0, 10.1) | 8.1 (–4.3, 22.4) | 6.1 (–12.2, 27.8) | –37.5 (–72.3, 25.2) | 14.4 (–11.7, 47.9) |
| ≥14 | 10.9 (–0.8, 24.0) | 9.0 (–3.0, 22.6) | **–25.6 (–42.1, –5.1)** * | 9.9 (–3.5, 25.5) | 4.1 (–15.1, 27.5) | –20.3 (–66.5, 74.3) | 32.1 (–0.1, 74.4) |
| **Use of cosmetics** | |  |  |  |  |  |  |
| 0 | Ref | Ref | Ref | Ref | Ref | Ref | Ref |
| 1~≤2 | 5.3 (–3.7, 15.0) | 4.6 (–4.9, 14.8) | 1.5 (–17.0, 24.7) | 1.5 (–8.8, 12.7) | –9.3 (–23.6, 7.8) | –9.1 (–49.3, 71.7) | 4.4 (–17.3, 32.0) |
| 3~<7 | –2.1 (–10.1, 6.6) | –1.3 (–9.8, 8.0) | 9.7 (–9.4, 33.3) | –0.8 (–10.3, 9.5) | 6.3 (–9.6, 25.2) | 64.5 (–14.7, 247.3) | –5.3 (–23.5, 17.5) |
| ≥7 | 3.2 (–4.4, 11.3) | 3.2 (–4.7, 11.8) | 1.3 (–14.5, 20.1) | 4.0 (–4.8, 13.7) | 3.6 (–10.3, 19.7) | –21.0 (–51.3, 29.8) | 14.7 (–5.8, 39.8) |
| **Dyeing or perming hair in past 3 months** | | |  |  |  |  |  |
| No | Ref | Ref | Ref | Ref | Ref | Ref | Ref |
| Yes | 2.2 (–5.7, 10.6) | –0.07 (–8.3, 8.7) | –8.7 (–23.2, 8.9) | 2.3 (–7.0, 12.3) | 3.0 (–11.5, 20.2) | –14.2 (–48.4, 50.7) | 12.1 (–8.8, 38.1) |

Note: Generalized linear regression models were adjusted for age, BMI, passive smoking, alcohol consumption, education level and income. Bold indicates that the item has statistical significance. **P* < 0.05.

Abbreviations: PCPs, personal care products; IVF, *in vitro* fertilization; ICSI, intracytoplasmic sperm injection; 2PN, two distinct pronuclei; BMI, body mass index; Ref, reference.

| **Supplementary** **Table S9.** Associations between PCPs characteristics and IVF/ICSI pregnancy outcomes among 1293 cycles with embryos transferred after excluding participants with endometriosis. | | | | |
| --- | --- | --- | --- | --- |
| **Characteristics**  (times per week) | Implantation | Clinical pregnancy | Miscarriage | Live birth |
|  | OR (95% CI) | OR (95% CI) | OR (95% CI) | OR (95% CI) |
| **Use of gel or soap** | |  |  |  |
| 0 | Ref | Ref | Ref | Ref |
| 1~<3 | 1.2 (0.71, 2.2) | 1.3 (0.76, 2.3) | 1.2 (0.47, 3.7) | 1.2 (0.71, 2.0) |
| 3~<7 | 1.2 (0.74, 2.0) | 1.5 (0.90, 2.4) | 0.89 (0.36, 2.5) | 1.4 (0.89, 2.3) |
| ≥7 | 1.1 (0.65, 1.7) | 1.1 (0.69, 1.8) | 0.62 (0.26, 1.7) | 1.2 (0.76, 1.9) |
| **Use of shampoo** | |  |  |  |
| ≤1 | Ref | Ref | Ref | Ref |
| 2~≤3 | 1.1 (0.64, 2.0) | 1.1 (0.61, 1.8) | 0.47 (0.19, 1.3) | 1.3 (0.77, 2.3) |
| 4~<7 | 1.1 (0.63, 1.8) | 1.1 (0.61, 1.8) | 0.50 (0.21, 1.3) | 1.3 (0.77, 2.2) |
| ≥7 | 1.2 (0.60, 2.4) | 0.96 (0.49, 1.9) | 0.49 (0.13, 1.7) | 1.2 (0.62, 2.3) |
| **Use of skin care products** | |  |  |  |
| 0 | Ref | Ref | Ref | Ref |
| 1~<7 | 0.84 (0.54, 1.3) | 0.96 (0.62, 1.5) | 0.59 (0.25, 1.4) | 1.1 (0.74, 1.7) |
| 7~<14 | 1.2 (0.80, 1.8) | 1.2 (0.80, 1.7) | 0.59 (0.29, 1.2) | 1.3 (0.92, 2.0) |
| ≥14 | 1.1 (0.73, 1.7) | 1.2 (0.81, 1.9) | 0.74 (0.35, 1.6) | 1.3 (0.87, 2.0) |
| **Use of cosmetics** | |  |  |  |
| 0 | Ref | Ref | Ref | Ref |
| 1~≤2 | 0.87 (0.61, 1.3) | 0.92 (0.64, 1.3) | 0.83 (0.36, 1.7) | 0.97 (0.69, 1.4) |
| 3~<7 | 0.88 (0.63, 1.2) | 0.98 (0.70, 1.4) | 1.3 (0.70, 2.5) | 0.92 (0.67, 1.3) |
| ≥7 | 0.95 (0.71, 1.3) | 1.0 (0.81, 1.4) | 0.89 (0.47, 1.6) | 1.1 (0.80, 1.4) |
| **Dyeing or perming hair in past 3 months** | | |  |  |
| No | Ref | Ref | Ref | Ref |
| Yes | 1.1 (0.83, 1.6) | 1.1 (0.91, 1.7) | 1.1 (0.65, 2.2) | 1.1 (0.86, 1.6) |

Note: Generalized linear regression models were adjusted for age, BMI, passive smoking, alcohol consumption, education level and income.

Abbreviations: PCPs, personal care products; IVF, *in vitro* fertilization; ICSI, intracytoplasmic sperm injection; OR, odd ratio; BMI, body mass index; Ref, reference.

| **Supplementary** **Table S10.** Associations between PCPs characteristics and IVF/ICSI pregnancy outcomes among 895 cycles with fresh embryos transferred after excluding participants with endometriosis. | | | | |
| --- | --- | --- | --- | --- |
| **Characteristics**  (times per week) | Implantation | Clinical pregnancy | Miscarriage | Live birth |
|  | OR (95% CI) | OR (95% CI) | OR (95% CI) | OR (95% CI) |
| **Use of gel or soap** | |  |  |  |
| 0 | Ref | Ref | Ref | Ref |
| 1~<3 | 0.56 (0.28, 1.1) | 0.89 (0.46, 1.7) | 1.1 (0.30, 4.4) | 0.91 (0.47, 1.8) |
| 3~<7 | 0.56 (0.28, 1.1) | 1.0 (0.55, 1.9) | 1.1 (0.37, 4.2) | 1.0 (0.56, 1.9) |
| ≥7 | 0.70 (0.37, 1.3) | 0.98 (0.55, 1.8) | 0.83 (0.28, 3.0) | 1.1 (0.59, 1.9) |
| **Use of shampoo** | |  |  |  |
| ≤1 | Ref | Ref | Ref | Ref |
| 2~≤3 | 1.2 (0.60, 2.3) | 1.2 (0.64, 2.5) | 0.91 (0.26, 4.3) | 1.3 (0.65, 2.6) |
| 4~<7 | 1.3 (0.69, 2.6) | 1.4 (0.74, 2.8) | 0.87 (0.25, 4.1) | 1.5 (0.76, 3.0) |
| ≥7 | 1.0 (0.45, 2.3) | 0.77 (0.33, 1.8) | 0.85 (0.13, 5.4) | 0.85 (0.36, 2.0) |
| **Use of skin care products** | |  |  |  |
| 0 | Ref | Ref | Ref | Ref |
| 1~<7 | 0.82 (0.48, 1.4) | 0.91 (0.54, 1.5) | 1.2 (0.44, 3.4) | 0.87 (0.52, 1.5) |
| 7~<14 | 0.96 (0.60, 1.5) | 0.95 (0.59, 1.5) | 0.73 (0.29, 2.0) | 1.0 (0.64, 1.6) |
| ≥14 | 0.78 (0.47, 1.3) | 0.72 (0.43, 1.2) | 1.3 (0.50, 3.8) | 0.69 (0.41, 1.1) |
| **Use of cosmetics** | |  |  |  |
| 0 | Ref | Ref | Ref | Ref |
| 1~≤2 | 0.91 (0.60, 1.4) | 0.95 (0.63, 1.4) | **2.3 (1.0, 5.0)** * | 0.76 (0.50, 1.2) |
| 3~<7 | 0.89 (0.60, 1.3) | 0.87 (0.59, 1.3) | **2.3 (1.0, 4.9)** * | 0.72 (0.48, 1.1) |
| ≥7 | 0.89 (0.63, 1.3) | 0.91 (0.65, 1.3) | 1.1 (0.50, 2.4) | 0.89 (0.63, 1.3) |
| **Dyeing or perming hair in past 3 months** | | |  |  |
| No | Ref | Ref | Ref | Ref |
| Yes | 1.1 (0.82, 1.7) | 1.1 (0.89, 1.9) | 1.1 (0.77, 3.0) | 1.1 (0.77, 1.6) |

Note: Generalized linear regression models were adjusted for age, BMI, passive smoking, alcohol consumption, education level and income. Bold indicates that the item has statistical significance. **P* < 0.05.

Abbreviations: PCPs, personal care products; IVF, *in vitro* fertilization; ICSI, intracytoplasmic sperm injection; OR, odd ratio; BMI, body mass index; Ref, reference.

| **Supplementary Table S11.** Associations between PCPs characteristics and IVF/ICSI parameters after excluding participants with male-factor infertility (n=1333). | | | | | | | | |
| --- | --- | --- | --- | --- | --- | --- | --- | --- |
| **Characteristics**  (times per week) | Number of retrieved oocytes | Number of mature oocytes | Maturation rate | | Number of  2PN zygotes | Fertilization rate | Cleavage rate | Blastocyst formation rate |
|  | % Change (95% CI) | % Change (95% CI) | | % Change (95% CI) | % Change (95% CI) | % Change (95% CI) | % Change (95% CI) | % Change (95% CI) |
| **Use of gel or soap** | |  | |  |  |  |  |  |
| 0 | Ref | Ref | | Ref | Ref | Ref | Ref | Ref |
| 1~<3 | –3.9 (–17.5, 12.3) | –6.4 (–20.0, 9.9) | | –6.3 (–34.0, 31.6) | -4.9 (-20.3, 13.9) | 6.9 (-19.0, 40.8) | 16.3 (-58.4, 197.6) | 19.7 (-18.5, 75.3) |
| 3~<7 | 0.14 (–12.6, 15.2) | –3.0 (–15.7, 12.1) | | –14.7 (–38.1, 15.6) | -0.70 (-15.1, 16.9) | 14.5 (-11.2, 47.1) | 59.3 (-40.1, 272.6) | 22.4 (-13.8, 73.0) |
| ≥7 | –4.7 (–16.4, 9.1) | –7.2 (–18.9, 6.8) | | –9.1 (–33.4, 21.9) | -5.4 (-18.7, 10.8) | 14.5 (-11.2, 47.1) | -2.3 (-61.1, 107.8) | -1.9 (-29.9, 36.5) |
| **Use of shampoo** | |  | |  |  |  |  |  |
| ≤1 | Ref | Ref | | Ref | Ref | Ref | Ref | Ref |
| 2~≤3 | 6.3 (–8.9, 24.6) | 1.9 (–13.0, 20.1) | | –10.8 (–37.0, 23.9) | 0.63 (-15.6, 20.8) | -2.3 (-26.6, 29.2) | 61.4 (-35.2, 253.2) | 12.4 (-23.0, 63.2) |
| 4~<7 | 2.7 (–11.6, 20.0) | –0.38 (–14.6, 17.0) | | –4.8 (–32.3, 31.2) | -2.3 (-17.6, 16.9) | 3.9 (-21.5, 36.4) | 77.9 (-27.2, 277.3) | 17.0 (-19.2, 68.5) |
| ≥7 | 1.4 (–16.0, 22.6) | –0.60 (–18.1, 21.0) | | 0.72 (–33.6, 51.9) | -6.3 (-24.6, 16.8) | -6.3 (-33.5, 31.4) | 2.0 (-63.1, 167.3) | 19.5 (-24.5, 89.1) |
| **Use of skin care products** | |  | |  |  |  |  |  |
| 0 | Ref | Ref | | Ref | Ref | Ref | Ref | Ref |
| 1~<7 | 7.4 (–5.2, 21.8) | 4.8 (–7.9, 19.5) | | –22.1 (–40.7, 1.8) | 11.2 (-3.8, 28.8) | 13.3 (-9.4, 41.5) | -23.0 (-69.3, 79.0) | 19.8 (-12.3, 63.5) |
| 7~<14 | 6.9 (–4.3, 19.8) | 6.0 (–5.5, 19.3) | | –6.5 (–27.1, 19.1) | 10.2 (-3.2, 25.9) | 15.0 (-5.5, 39.7) | -35.8 (-72.4, 31.8) | 25.7 (-4.6, 65.5) |
| ≥14 | 10.2 (–2.1, 24.3) | 8.3 (–4.3, 22.7) | | **–23.0 (–40.7, –0.6)** * | 10.1 (-4.2, 26.9) | 9.3 (-11.6, 34.8) | -24.3 (-68.9, 67.1) | **37.5 (2.2, 84.9)** * |
| **Use of cosmetics** | |  | |  |  |  |  |  |
| 0 | Ref | Ref | | Ref | Ref | Ref | Ref | Ref |
| 1~≤2 | 2.6 (–6.8, 12.8) | 3.0 (–6.8, 13.7) | | 6.3 (–14.0, 32.1) | 0.94 (-9.8, 12.7) | -5.6 (-21.0, 13.1) | 2.8 (-43.6, 98.4) | 15.8 (-9.7, 48.8) |
| 3~<7 | –1.6 (–10.4, 7.8) | –0.60 (–9.8, 9.4) | | 11.8 (–8.8, 37.8) | 1.6 (-8.7, 13.0) | 12.5 (-5.3, 34.1) | 64.7 (-15.3, 251.6) | 2.3 (-18.9, 29.2) |
| ≥7 | 4.1 (–4.0, 12.9) | 5.1 (–3.4, 14.3) | | 9.1 (–8.8, 30.7) | 6.1 (-3.4, 16.5) | 4.9 (-9.7, 21.9) | -14.4 (-47.6, 41.9) | 19.3 (-3.2, 47.2) |
| **Dyeing or perming hair in past 3 months** | | | |  |  |  |  |  |
| No | Ref | Ref | | Ref | Ref | Ref | Ref | Ref |
| Yes | 1.3 (–7.0, 10.3) | –0.49 (–9.1, 8.8) | | –5.3 (–21.1, 14.1) | 0.56 (-9.1, 11.0) | -2.1 (-16.3, 14.8) | -21.2 (-52.2, 36.4) | 1.2 (-18.6, 26.1) |

Note: Generalized linear regression models were adjusted for age, BMI, passive smoking, alcohol consumption, education level and income. Bold indicates that the item has statistical significance. **P* < 0.05.

Abbreviations: PCPs, personal care products; IVF, *in vitro* fertilization; ICSI, intracytoplasmic sperm injection; 2PN, two distinct pronuclei; BMI, body mass index; Ref, reference.

| **Supplementary** **Table S12.** Associations between PCPs characteristics and IVF/ICSI pregnancy outcomes among 1210 cycles with embryos transferred after excluding participants with male-factor infertility. | | | | |
| --- | --- | --- | --- | --- |
| **Characteristics**  (times per week) | Implantation | Clinical pregnancy | Miscarriage | Live birth |
|  | OR (95% CI) | OR (95% CI) | OR (95% CI) | OR (95% CI) |
| **Use of gel or soap** | |  |  |  |
| 0 | Ref | Ref | Ref | Ref |
| 1~<3 | 1.3 (0.72, 2.3) | 1.4 (0.82, 2.5) | 1.1 (0.40, 3.3) | 1.3 (0.77, 2.3) |
| 3~<7 | 1.4 (0.82, 2.3) | 1.6 (0.98, 2.7) | 0.79 (0.32, 2.3) | 1.6 (0.98, 2.7) |
| ≥7 | 1.1 (0.66, 1.8) | 1.1 (0.69, 1.9) | 0.57 (0.23, 1.6) | 1.3 (0.79, 2.1) |
| **Use of shampoo** | |  |  |  |
| ≤1 | Ref | Ref | Ref | Ref |
| 2~≤3 | 1.1 (0.62, 1.9) | 1.0 (0.58, 1.8) | 0.44 (0.18, 1.2) | 1.3 (0.75, 2.1) |
| 4~<7 | 1.1 (0.61, 1.9) | 1.0 (0.59, 1.8) | 0.46 (0.20, 1.2) | 1.3 (0.76, 2.3) |
| ≥7 | 1.1 (0.56, 2.3) | 0.94 (0.47, 1.9) | 0.57 (0.17, 1.9) | 1.1 (0.58, 2.3) |
| **Use of skin care products** | |  |  |  |
| 0 | Ref | Ref | Ref | Ref |
| 1~<7 | 0.91 (0.57, 1.4) | 1.0 (0.7, 1.6) | 0.53 (0.22, 1.3) | 1.2 (0.80, 1.9) |
| 7~<14 | 1.2 (0.82, 1.8) | 1.2 (0.8, 1.8) | 0.54 (0.26, 1.2) | 1.4 (0.96, 2.1) |
| ≥14 | 1.2 (0.74, 1.8) | 1.2 (0.8, 1.9) | 0.66 (0.31, 1.5) | 1.3 (0.88, 2.1) |
| **Use of cosmetics** | |  |  |  |
| 0 | Ref | Ref | Ref | Ref |
| 1~≤2 | 0.87 (0.60, 1.3) | 0.86 (0.60, 1.2) | 0.73 (0.30, 1.6) | 0.95 (0.66, 1.3) |
| 3~<7 | 0.96 (0.68, 1.4) | 1.1 (0.76, 1.5) | 1.5 (0.76, 2.7) | 0.96 (0.69, 1.4) |
| ≥7 | 1.1 (0.84, 1.6) | 1.2 (0.90, 1.7) | 0.74 (0.38, 1.4) | 1.3 (0.94, 1.7) |
| **Dyeing or perming hair in past 3 months** | | |  |  |
| No | Ref | Ref | Ref | Ref |
| Yes | 1.1 (0.74, 1.4) | 1.1 (0.84, 1.6) | 1.1 (0.60, 2.1) | 1.1 (0.81, 1.5) |

Note: Generalized linear regression models were adjusted for age, BMI, passive smoking, alcohol consumption, education level and income.

Abbreviations: PCPs, personal care products; IVF, *in vitro* fertilization; ICSI, intracytoplasmic sperm injection; OR, odd ratio; BMI, body mass index; Ref, reference.

| **Supplementary** **Table S13** Associations between PCPs characteristics and IVF/ICSI pregnancy outcomes among 827 cycles with fresh embryos transferred after excluding participants with male-factor infertility. | | | | |
| --- | --- | --- | --- | --- |
| **Characteristics**  (times per week) | Implantation | Clinical pregnancy | Miscarriage | Live birth |
|  | OR (95% CI) | OR (95% CI) | OR (95% CI) | OR (95% CI) |
| **Use of gel or soap** | |  |  |  |
| 0 | Ref | Ref | Ref | Ref |
| 1~<3 | 0.54 (0.25, 1.1) | 0.94 (0.46, 1.9) | 0.83 (0.23, 3.5) | 1.0 (0.51, 2.1) |
| 3~<7 | 0.62 (0.31, 1.2) | 1.1 (0.57, 2.1) | 0.91 (0.29, 3.5) | 1.2 (0.60, 2.3) |
| ≥7 | 0.64 (0.32, 1.2) | 1.0 (0.54, 1.9) | 0.79 (0.26, 3.0) | 1.1 (0.60, 2.2) |
| **Use of shampoo** | |  |  |  |
| ≤1 | Ref | Ref | Ref | Ref |
| 2~≤3 | 1.1 (0.53, 2.2) | 1.1 (0.56, 2.3) | 0.87 (0.24, 4.1) | 1.2 (0.58, 2.5) |
| 4~<7 | 1.3 (0.65, 2.6) | 1.4 (0.70, 2.8) | 0.74 (0.21, 3.5) | 1.5 (0.74, 3.1) |
| ≥7 | 1.1 (0.48, 2.6) | 0.78 (0.34, 1.8) | 1.0 (0.18, 6.1) | 0.82 (0.34, 2.0) |
| **Use of skin care products** | |  |  |  |
| 0 | Ref | Ref | Ref | Ref |
| 1~<7 | 0.94 (0.53, 1.7) | 0.97 (0.56, 1.7) | 1.4 (0.49, 4.4) | 0.90 (0.51, 1.6) |
| 7~<14 | 0.91 (0.55, 1.5) | 0.93 (0.57, 1.5) | 0.97 (0.37, 2.9) | 0.95 (0.58, 1.6) |
| ≥14 | 0.87 (0.51, 1.5) | 0.81 (0.48, 1.4) | 1.4 (0.50, 4.2) | 0.76 (0.45, 1.3) |
| **Use of cosmetics** | |  |  |  |
| 0 | Ref | Ref | Ref | Ref |
| 1~≤2 | 0.97 (0.63, 1.5) | 1.0 (0.68, 1.6) | **2.3 (1.0, 5.0)** * | 0.81 (0.53, 1.2) |
| 3~<7 | 1.0 (0.67, 1.6) | 0.99 (0.65, 1.5) | **2.4 (1.1, 5.4)** * | 0.78 (0.51, 1.2) |
| ≥7 | 0.87 (0.61, 1.2) | 0.97 (0.68, 1.4) | 1.2 (0.54, 2.7) | 0.93 (0.64, 1.3) |
| **Dyeing or perming hair in past 3 months** | | |  |  |
| No | Ref | Ref | Ref | Ref |
| Yes | 1.1 (0.75, 1.6) | 1.1 (0.88, 1.9) | 1.1 (0.71, 2.8) | 1.1 (0.77, 1.7) |

Note: Generalized linear regression models were adjusted for age, BMI, passive smoking, alcohol consumption, education level and income. Bold indicates that the item has statistical significance. **P* < 0.05.

Abbreviations: PCPs, personal care products; IVF, *in vitro* fertilization; ICSI, intracytoplasmic sperm injection; OR, odd ratio; BMI, body mass index; Ref, reference.


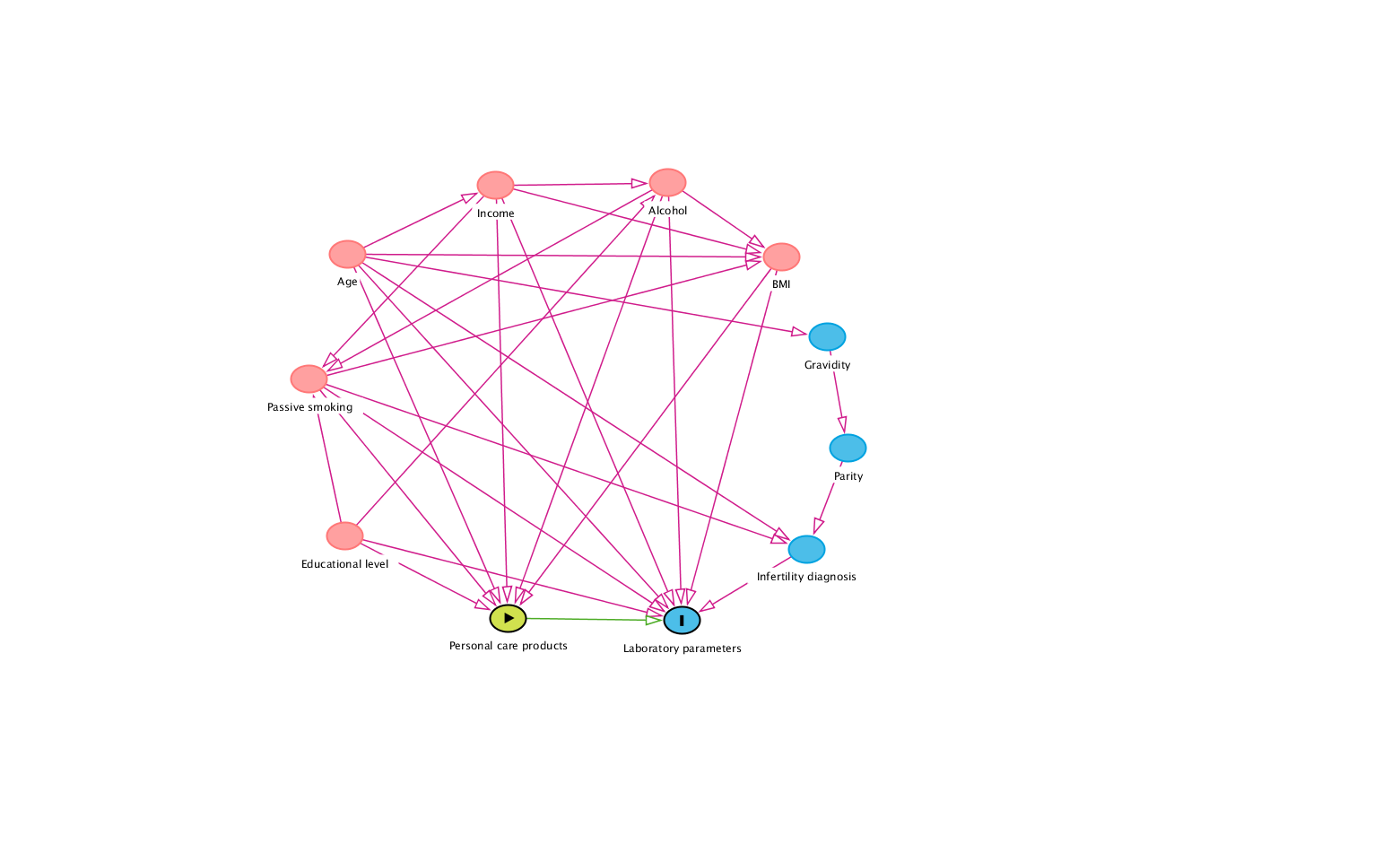


**Supplementary** **Figure S1.** Directed acyclic graph of intermediate IVF/ICSI outcomes.


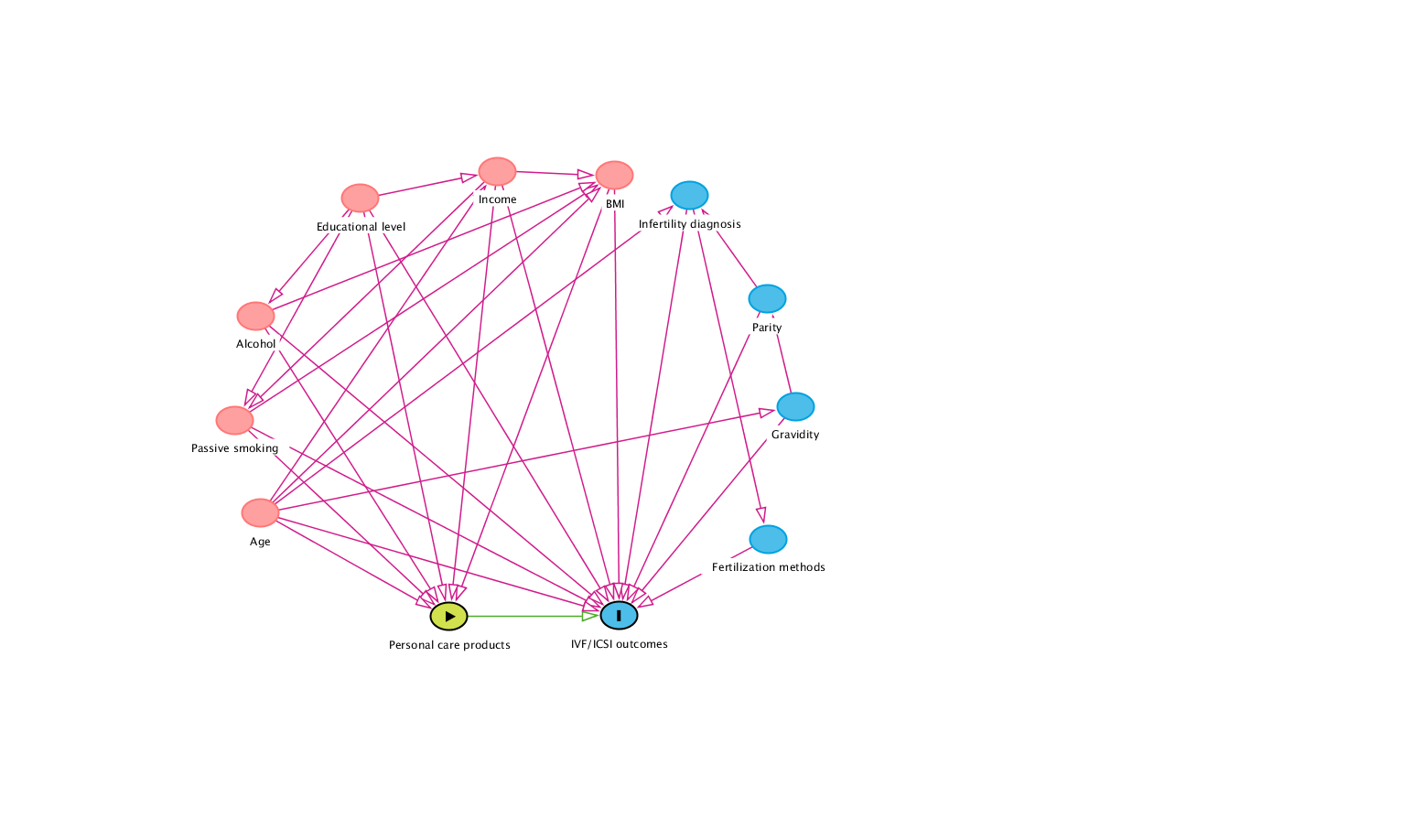


**Supplementary** **Figure S2.** Directed acyclic graph of early pregnancy outcomes.
